# The Role of A-Kinase Anchoring Proteins for Inhibitory cAMP Signalling in Platelets

**DOI:** 10.3390/cells15060553

**Published:** 2026-03-19

**Authors:** Shannon Barkey, Albert Smolenski

**Affiliations:** UCD Conway Institute, School of Medicine, University College Dublin, Belfield, D04V1W8 Dublin, Ireland; shannon.barkey@ucdconnect.ie

**Keywords:** endothelium, prostacyclin, cAMP, PKA, AKAP, phosphodiesterase

## Abstract

**Highlights:**

**What are the main findings?**

- A-kinase anchoring proteins (AKAP) are expressed in human platelets.
- AKAPs provide spatial and temporal coordination of cAMP signalling and platelet inhibition.

**What are the implications of the main findings?**

- AKAPs fine-tune endothelium-dependent platelet regulation.
- AKAPs could be new therapeutic targets that prevent thrombus formation through endogenous cAMP signalling.

**Abstract:**

Platelets are small circulating blood cells that mediate haemostasis and thrombosis. Platelets respond to vascular damage by adhesion, granule release, and aggregation. Healthy endothelial cells inhibit platelets through prostacyclin-induced cAMP signalling. Intracellular cAMP activates protein kinase A (PKA), a tetrameric kinase composed of two regulatory (R) and two catalytic (C) subunits. cAMP-binding triggers dissociation of C subunits from the PKA complex and phosphorylation of substrate proteins, which mediate platelet inhibition. The R subunits of PKA are known to be attached to A-kinase anchoring proteins (AKAPs), which enable subcellular compartmentalisation of cAMP signalling. Proteomics have identified 22 AKAPs in platelets, but only a few of these have been studied in detail. This review summarises current knowledge about platelet AKAPs, including studies done regarding other cells. Possible integration of AKAPs into platelet signalling is explored with a focus on subcellular localisation, interaction partners, and PKA-mediated substrate phosphorylation. As main platelet compartments, the plasma membrane, endosomes, mitochondria, the Golgi, the dense tubular system, and the cytoskeleton are considered. Potential roles of individual AKAPs in platelet inhibition are discussed, and open questions in the field are defined.

## 1. Introduction

Platelets are small blood cells that respond rapidly to vascular injury through adhesion, granule secretion, and aggregation [1]. Thrombosis underlies many cardiovascular disease-related deaths globally, which is driven by dysregulated platelet activation. Central to preventing excessive activation is cyclic adenosine monophosphate (cAMP), a potent endogenous inhibitor of platelet function, which acts primarily by activating protein kinase A (PKA) to suppress many aspects of platelet activation, including calcium mobilisation, granule release and integrin activation. cAMP is synthesised by adenylyl cyclases (ACs) in response to the binding of endothelium-derived prostacyclin (PGI_2_) to platelet IP receptors. cAMP levels are further controlled by phosphodiesterases (PDEs), which degrade cAMP. Platelets express multiple AC and PDE isoforms together with PKA subtypes I and II and somehow coordinate these components to inhibit specific functions without compromising essential haemostasis [2,3,4]. This specificity is most likely supported by spatial compartmentalisation of cAMP signalling. cAMP is organised into nanodomains where PKA activity is focused onto individual substrates by A-kinase anchoring proteins (AKAPs) [5]. Recent literature has shown that PKA regulatory subunits cooperate with AKAPs and PDEs to sharpen cAMP gradients induced by receptor signalling [6,7].

This review comprehensively examines the AKAPs identified in human platelets through proteomic, biochemical and functional studies. We evaluate the potential role of individual AKAPs in guiding PKA to its substrates and coordinating cAMP-mediated control of platelet function. Developing a clearer understanding of AKAPs and their role in platelet signalling may yield novel anti-thrombotic strategies [8,9].

## 2. Platelet Function and Signalling

### 2.1. Overview of Platelet Structure and Function

Platelets are anucleate, discoid cells (2–5 µm) that are derived from megakaryocytes, which are primarily located in the bone marrow. Each adult human contains around one trillion platelets in the vasculature, with an 8–10-day turnover [10]. Giulio Bizzozero was the first to describe platelets as another element of blood aside from erythrocytes and leukocytes [11,12]. The platelet architecture can be divided into a peripheral zone rich in surface glycoproteins (for example, GPIb-IX-V and integrin α_IIb_β_3_), a sol–gel zone containing the contractile cytoskeleton, and an organelle zone housing alpha (50–80 per platelet) and dense (or delta) granules (3–8 per platelet) that store adhesive and signalling molecules [1].

The sol–gel zone includes a circumferential microtubule coil that maintains the discoid shape and an actin-based contractile cytoskeleton that drives shape change and granule centralization during activation. The organelle zone also contains mitochondria that provide energy for these processes, and rudiments of Golgi complexes and endoplasmic reticulum that may contribute to protein synthesis in less than 1% of platelets [13]. The open canalicular system (OCS) is an invagination of the plasma membrane inward, forming tubule conduits. The OCS is also considered an entry site for platelet endocytosis. This allows for the uptake of plasma proteins such as fibrinogen via receptor-mediated mechanisms through integrin α_IIb_β_3_, which is then transported through multivesicular bodies (MVB) and internalised into alpha granules for storage.

Platelets use both clathrin-dependent and clathrin-independent endocytic pathways to modulate surface receptor expression (α_IIb_β_3_ and P2Y12) and regulate signalling [13,14]. Dense granules are lysosome-related organelles originating from MVBs and contain Ca^2+^ ions and small molecules like ADP, which are released into the extracellular space during platelet activation [15]. The dense tubular system (DTS) of platelets is related to the sarcoplasmic or endoplasmic reticulum in other cells, containing Ca^2+^ ions that are released into the cytosol following receptor activation [16].

Platelets play a key role in haemostasis. Their primary function is arresting bleeding by adhering to injured vessel walls and forming aggregates that seal the breached vasculature. Upon activation, platelets can undergo shape change, spread on adhesive vascular surfaces, and secrete granule contents that support coagulation and amplify the hemostatic responses. However, platelets’ contribution expands beyond haemostasis to several secondary roles. Platelets contribute to inflammation through P-selectin expression and proinflammatory factor release, participate in antimicrobial host defence, influence tumour growth and metastasis through adhesive and secretory mechanisms, and are required for blood-lymphatic vessel separation [17,18,19].

### 2.2. Platelet Activation

When vascular injury occurs, platelets respond rapidly through adhesion, activation and aggregation (primary haemostasis). The platelets can tether to the exposed subendothelial matrix via GPIb-IX-V binding to von Willebrand factor and collagen [2]. This is followed by further adhesion through collagen receptors such as GPVI and integrin α_2_β_1_, which generate early outside-in activation signals. In parallel, thrombin, which is generated by the coagulation cascade, activates platelets through protease-activated receptors (PARs) [20].

PARs are G-protein-coupled receptors (GPCRs) which, together with the tyrosine kinase-linked GPVI receptor, initiate a series of converging intracellular pathways [21]. Central to these pathways is the activation of phospholipase C, which hydrolyses phosphatidylinositol 4,5-biphosphate to generate inositol 1,4,5-triphosphate (IP_3_) and diacylglycerol (DAG). IP_3_ stimulates the rapid release of Ca^2+^ ions from the DTS, while DAG activates protein kinase C (PKC) [22]. Ca^2+^ mobilisation and PKC signalling drive the cytoskeletal rearrangements required for platelet shape change, promote granule secretion and induce inside-out signalling that converts integrin α_IIb_β_3_ to its high-affinity conformation [3,23]. Simultaneously, G_12/13_-mediated activation of the small G protein RhoA contributes to actomyosin contraction and further supports cytoskeletal remodelling.

As the platelets activate, they release secondary mediators, including ADP from dense granules as well as thromboxane A2, which reinforce activation through positive feedback loops by engaging their respective GPCRs (P2Y1 and P2Y12 receptors for ADP; TP receptor for thromboxane A2) [24]. This amplifies integrin activation, fibrinogen binding, and platelet-platelet cohesion, thus promoting rapid thrombus propagation [25]. As the platelets activate, α_IIb_β_3_ transforms to a high-affinity state, which allows fibrinogen, fibrin and VWF binding to form bridges between the platelets and promote aggregation [26]. Fibrin fibres form a stabilising scaffold around the accumulated platelet mass, and the thrombus is further consolidated by platelet-mediated clot retraction [3,23].

### 2.3. Platelet Inhibitory Pathways

Apart from the cyclic nucleotide systems discussed below, platelets express immunoreceptor tyrosine-based inhibition motif (ITIM)-containing receptors, which can induce inhibitory signalling affecting tyrosine kinase pathways primarily. The most prominent of these are the platelet endothelial cell adhesion molecule-1 (PECAM-1, CD31) and G6b-B receptors [4,27]. PECAM-1 inhibits GPVI-mediated platelet activation by collagen. In particular, tyrosine phosphorylation of PECAM-1 ITIMs leads to recruitment of SH2 domain-containing lipid or protein tyrosine phosphatases (Shp) which dephosphorylate the immunoreceptor tyrosine-based activation motif (ITAM)-containing collagen receptor complex GPVI-Fc receptor (FcR) γ-chain, leading to reduced collagen-mediated platelet activation. Similarly, G6b-B recruits Shp 1 and 2 to downregulate ITAM receptor signalling, including GPVI and CLEC2 receptor signalling. Platelets also express a few other ITIM-containing receptors at lower levels [27,28]. GPVI as well as GPIb-IX-V receptor signalling appear not to be major targets of cyclic nucleotide-dependent platelet inhibition [29]. Instead, cAMP and cGMP pathways mainly block GPCR responses and common downstream signals involving Ca^2+^, G proteins, and the cytoskeleton.

## 3. Overview of cAMP Signalling in Platelets

### 3.1. cAMP, a Key Inhibitory Pathway

Cyclic adenosine monophosphate (cAMP) serves as the most potent endogenous inhibitor of platelet activation and plays a critical role in maintaining the balance between haemostasis and thrombosis. Intact endothelium releases PGI_2_ and nitric oxide (NO), which act through distinct cyclic nucleotide pathways. PGI_2_ stimulates AC to increase intracellular cAMP, while NO activates guanylyl cyclase to raise cGMP levels. Within platelets, elevated cAMP activates PKA and cGMP activates cGMP-dependent protein kinase (PKG), which phosphorylates a broad spectrum of substrate proteins. These phosphorylated substrates suppress multiple platelet functions, including Ca^2+^ mobilisation, G protein activation, shape change, granule secretion, integrin activation, and adhesion.

**Figure 1:**
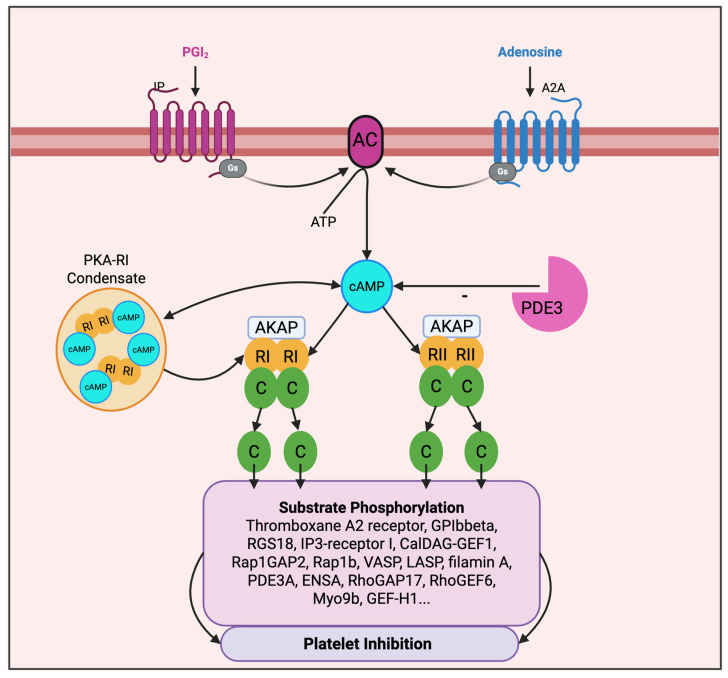
Overview of prostacyclin- and adenosine-stimulated cAMP/PKA signalling in platelets. Prostacyclin (PGI_2_)- or adenosine- dependent activation of Gs-coupled IP or A2A receptors elevates cAMP, activating cAMP-dependent protein kinase (PKA) and suppressing platelet activation pathways. Phosphodiesterase type 3A (PDE3A) is the major platelet PDE mediating cAMP hydrolysis and contributing to cAMP compartmentalisation. A-kinase anchoring proteins (AKAP) generate local PKA signalling compartments, facilitating substrate phosphorylation. PKA is a tetramer of two regulatory (R) and two catalytic (C) subunits. Phase separation of PKA-RI subunits might provide an additional regulatory layer by forming biomolecular condensates that sequester and concentrate cAMP with potential impact on local cAMP gradients and availability of C subunits. C subunits detach from cAMP-bound R subunits to phosphorylate numerous substrate proteins leading to platelet inhibition. Created with BioRender. Smolenski, A. (2026) https://BioRender.com/zay7ubh.

cAMP levels are controlled by the balance between the synthesis by AC and the degradation by PDEs (Figure 1). Upon activation of G_s_-coupled receptors (PGI_2_ IP and adenosine A2A and possibly A2B receptors) [30,31,32], AC catalyses the conversion of ATP to cAMP. Platelets express AC3, AC5 and AC6, though the specific functional roles of each isoform remain to be elucidated [2,5].

Recent evidence obtained in cells other than platelets indicates that cAMP generation might not always be exclusively confined to the plasma membrane. G protein-coupled receptors (GPCRs) can localise to and signal from intracellular compartments such as endosomes and the Golgi apparatus, generating distinct cAMP pools that regulate specific cellular functions. Internalised receptors can associate with ACs and AKAPs to form receptor-associated independent cAMP nanodomains (RAINs) [6]. Indirect evidence for plasma membrane-independent cAMP formation in platelets comes from Förster resonance energy transfer (FRET) biosensor studies showing PKA activation within forming thrombi, showing spatial heterogeneity in cyclic nucleotide signalling [33]. Similarly, cGMP sensor studies showed shear-dependent NO-induced cGMP signalling suppresses Ca^2+^ signalling at the thrombus periphery, promoting thrombus dissolution [34]. However, the spatio-temporal resolution of these studies was not sufficient to determine cyclic nucleotide signals inside single platelets. A recent study by Webb et al. provided evidence that AC6 is the dominant isoform in both human and mouse platelets. Loss of AC6 selectively impaired PKA-dependent inhibition of PAR-mediated activation without affecting GPVI responses [35].

cAMP degradation is mediated by PDE2 and 3 through the hydrolysis of the 3′-phosphoester bond, leading to the formation of 5′-AMP. PDE3 is the most prominent regulator of cAMP levels in platelets [36,37]. PDE3 is inhibited by cGMP, whereas PDE2 is stimulated by cGMP. Platelets also express PDE5, which is cGMP-specific [38].

### 3.2. Protein Kinase A

PKA is the principal effector of cAMP in platelets and functions as a brake on activation. The holoenzyme is composed of a dimer of regulatory (R) subunits bound to two catalytic (C) subunits, forming type I (RIɑ/β) and type II (RIIɑ/β) isoforms that differ in subcellular localisation and substrate preference [2,5,39]. One mechanism for controlling PKA might be a molar excess of R subunits relative to C subunits [40]. Quantitative analysis has indicated a skewed stoichiometry with R subunit isoforms present at high concentrations and in about 10-fold molar excess over C subunits in many cells and tissues [41,42]. This stoichiometry might indicate a high overall buffering capacity for cAMP, which ensures the rapid recapture of liberated C subunits, preventing their unrestrained activity or “free swimming” following activation [40]. Platelet proteomic data currently suggest only slightly higher expression levels of R over C subunits in platelets: RIɑ 7876, RIβ 2635, RIIɑ 3479, RIIβ 7633 (total R 21623); Cɑ 8713, Cβ 9615 (total C 18328) copies per platelet [43]. Active PKA C subunits have also been shown to interact with the NFkB complex in platelets, providing another mechanism of cAMP-independent PKA activation, which could be involved in negative feedback following exposure of platelets to thrombin or collagen [44].

An additional layer of PKA regulation is provided by PDEs. cAMP buffering PDEs, which have low catalytic rates, can generate 10–60 nm low cAMP nanodomains that protect local PKA from activation. Agonist-stimulated cAMP synthesis is thought to lead to flooding of these nanodomains, producing rapid, spatially confined phosphorylation events mediated by PKA [45]. Complementary work showed that the RIɑ and RIβ regulatory subunits might undergo liquid–liquid phase separation [46,47]. These RI condensates sequester cAMP and might contribute to compartmentalisation of cAMP signalling (Figure 1). Disrupting RI phase separation permits unrestrained signalling [47]. The role of RI phase separation for cAMP signalling in platelets has not been studied.

The PKA holoenzyme is kept inactive by the regulatory subunits, whose inhibitory domains occupy the catalytic pocket of the C subunits. In type-I isoforms, RI subunits act as a non-phosphorylatable pseudosubstrate with the consensus sequence RRxA/G, directly blocking substrate access without undergoing phosphorylation. In contrast, type II isoforms contain a phosphorylatable inhibitory serine (pS112) on RII that is already phosphorylated in the resting holoenzyme, resulting in a “single-turnover” autophosphorylation that weakens R-C affinity and primes the complex for activation. The binding of cAMP to the R subunits triggers the conformational opening that exposes the phosphorylated RII epitope (or the RI pseudosubstrate) and releases active C subunits. Termination of the signal then requires phosphatase-mediated dephosphorylation of the RII inhibitory site, allowing for rapid reassociation of R and C subunits. This dual mechanism, pseudosubstrate inhibition for RI and pre-phosphorylated substrate inhibition for RII, regulates both activation and inactivation of PKA signalling [48].

Protein kinase A targets a wide range of substrate proteins across various subcellular compartments in platelets to achieve rapid inhibition of activation, adhesion and aggregation. At the plasma membrane, PKA activates GTPase-activating proteins (GAPs) such as RGS18 (via phosphorylation on serine 216) or Rap1GAP2 (on serine 7), which dampens heterotrimeric Gɑq/Gɑi and Rap1 signalling, thereby blocking Ca^2+^ signalling, integrin α_IIb_β_3_ activation, and aggregation [49,50,51]. Simultaneously, PKA phosphorylates and inhibits guanine-nucleotide exchange factors like the Rap1-specific CalDAG-GEFI (on serine 587) and regulates membrane receptors such as the thromboxane A2 receptor and GPIbβ subunits, while also causing Rap1b to redistribute from the membrane to the cytosol. Within the cytoplasm and at internal membranes like the DTS, PKA prevents the essential rise in cytosolic calcium (Ca^2+^) by phosphorylating the IP_3_ receptor, thus blocking Ca^2+^ release [52]. PKA also controls signalling homeostasis by activation of PDE3A through phosphorylation of serine 312, thus initiating a negative feedback loop for cAMP. Furthermore, PKA dictates cytoskeletal dynamics by targeting highly abundant actin-binding proteins like vasodilator-stimulated phosphoprotein (VASP, on serines 157 and 239). VASP has been shown to be involved in PKA and PKG-dependent platelet inhibition and might play a role in the activation of Rap1 [51]. LASP (LIM and SH3 domain protein) is another cytoskeletal protein phosphorylated (on serine 146) by PKA, which reduces its binding to F-actin and focal adhesions [53]. The actin-controlling G proteins Rac1 and RhoA are regulated by PKA-mediated phosphorylation of their GAPs (RhoGAP17, Myo9b) and GEFs (RhoGEF6, GEF-H1) [54,55]. The regulatory subunit of myosin-light-chain phosphatase, MYPT1, can be phosphorylated by PKA and activation of PKA can inhibit RhoA signalling, thereby preserving myosin light chain phosphatase activity in platelets [56]. However, there is currently no direct evidence of PKA phosphorylation of MYPT1 in platelets [57].

Platelet phosphoproteomics carried out by Beck et al. identified 137 phosphopeptides with a PKA consensus motif, expanding the known substrate list beyond the classic G protein and actin-related targets. These newly suggested PKA substrates include vesicle-associated proteins such as SCAMP3, membrane junction components like claudin-5, and many kinases. Rapidly phosphorylated low molecular weight protein phosphatase 2A inhibitory proteins ENSA and ARPP19 were also detected and later confirmed by further studies [58]. Additional candidates with putative PKA sites include adaptor and ubiquitin-related proteins such as Cullin4A, CYLD, and UBE2O, none of which have been functionally characterised in platelet inhibition [59].

Subcellular compartmentalisation of cAMP/PKA signalling is enabled by AKAPs, which are likely to direct subsets of PKA to specific substrates and sites of action.

## 4. A-Kinase Anchoring Proteins: General Structure and Roles

A-kinase anchoring proteins (AKAPs) constitute a structurally diverse family of over 50 human scaffolding proteins that are essential for the spatial and temporal specificity of cAMP signalling, ensuring that this pathway mediates specific biological effects [60]. AKAPs achieve compartmentalisation by binding the PKA holoenzyme and restricting its action to localised “signalling islands”, typically within 200–400 nm of the anchoring site [61]. Structurally, all functional AKAPs have two key parts. They have a unique targeting domain that anchors the complex to specific subcellular locations like the membrane, or cytoskeleton and a conserved A-kinase binding (AKB) domain [60,61,62]. The AKB is an amphipathic helix, generally 14–18 residues long, which interacts with the N-terminal docking and dimerization (D/D) domain of the PKA regulatory (R) subunit homodimer [61]. AKAPs often preferentially anchor either Type I or Type II PKA subunits with high nanomolar affinity (K_D_ = 1–5 nM), although dual-specificity AKAPs also exist.

Type II PKA is the predominant isoform that interacts with AKAPs [62,63]. The anchoring of Type II PKA (RII) relies on hydrophobic interactions within a preformed groove on the RII D/D domain. Using AKAP-IS, a short synthetic peptide that was engineered to bind selectively to type-II regulatory subunits of PKA, Gold et al. could show that the high-affinity binding of AKAP-IS to RII was abolished when bulky residues were introduced at positions 9, 13 or 16 of the AKAP helix, confirming that a tightly packed hydrophobic core is essential for RII selectivity [64]. Kinderman’s high-resolution structural analysis of AKAP10 (D-AKAP2) binding to RII reinforced this view by demonstrating that the binding of the AKAP amphipathic helix induced crucial asymmetry in the RII D/D domain [63]. This interaction was found to be mediated by an “induced” fit mechanism, whereby upon AKAP docking, only the flexible N-terminal segment of one RII protomer was recruited and stabilised at the binding site, while the N-terminus of the second protomer remained disordered. This stabilisation was critical for the high-affinity interaction. The stabilised N-terminus was shown to anchor to the AKAP peptide through essential hydrophobic contacts [63]. This binding mechanism was shown to dictate isoform selectivity between Type II and Type I PKA. A key selectivity determinant was identified within the stable hydrophobic surface that exhibited a strong preference for smaller residues, such as valine 13 on the AKAP. In contrast, the RIa D/D domain groove was shown to accept larger bulky side chains, such as tryptophan at this position. This preference by the RIIa D/D domain for a specific residue contributed significantly to its ability to discriminate between RI and RII isoforms, enabling precise control over anchoring events [63]. Collectively, structural studies [63,64], evolutionary analyses and systematic motif mapping [65,66] have established that PKA isotype selectivity for AKAPs is governed by a combination of a (i) a hydrophobic core that fits the shape of the RII groove, (ii) steric constraints at position 13 of the AKAP helix that discriminates between the flat RII surface and the more accommodating RI surface, and (iii) conserved sequence motifs such as the FA motif (phenylalanine and alanine) at positions 1 and 2 of the AKB that bias binding toward RI [63,64,65,66]. Of note, a few proteins have been described that can bind R subunits by AKB-independent mechanisms [67].

In addition to anchoring PKA, AKAPs function as multivalent scaffolds by anchoring other signalling components such as PDEs, phosphatases and small G proteins into the same complex. These scaffolding capabilities allow for integrated signal transmission by coupling the initiation of the cAMP signal with its rapid termination [5,68,69].

## 5. AKAP Expression in Platelets

Recent proteomics studies have confirmed the expression of many AKAPs in human platelets. The AKAPs discussed in this paper have been selected based on Huang et al., a recent compilation and integration of megakaryocyte and platelet transcriptome and proteome data from various sources, which provides estimates on protein expression levels in copies/platelet [43]. As a second source, a study using cAMP beads to enrich platelet AKAPs was used [70]. AKAPs were sorted according to (i) subcellular localisation, and (ii) expression levels, starting with highly expressed proteins (Table 1). Possible links to PKA substrates and platelet functions are discussed, although current knowledge on AKAPs is mostly based on studies written on cells other than platelets.

### 5.1. Plasma Membrane AKAPs

**Figure 2:**
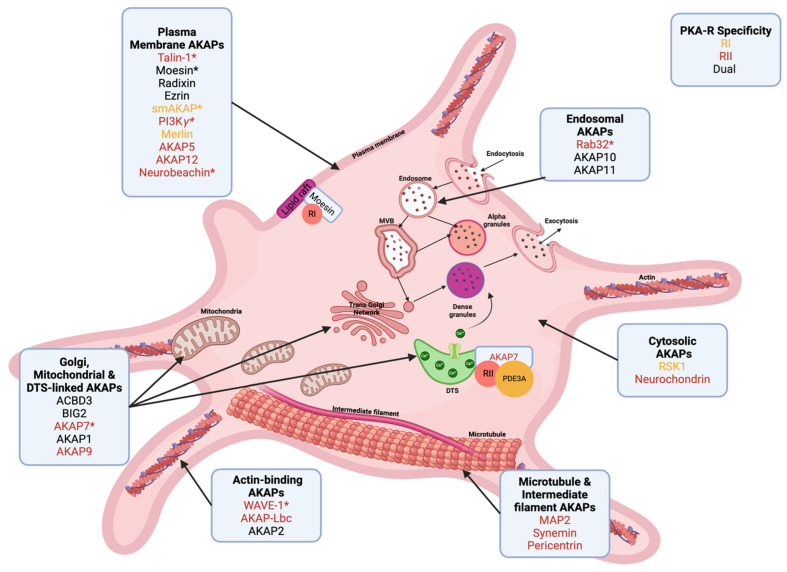
Predicted subcellular location of AKAPs in human platelets. Schematic representation of a human platelet highlighting the predicted subcellular localisation of AKAPs identified by platelet proteomic analyses [43,70]. AKAPs for which platelet-specific experimental evidence exists are indicated as confirmed components of platelet signalling complexes [75,83]. Moesin has been characterised as an RI-selective AKAP that co-localises with PKA-RI in plasma membrane lipid rafts. AKAP7δ formed a cytosolic signalling complex with PKA-RII and PDE3A. Although AKAP7 has not been experimentally linked to a specific platelet organelle, its established role in calcium-regulatory compartments in other cell types supports a potential association with the dense tubular system (DTS). * marks proteins that have been studied in platelets in general, in addition to moesin and AKAP7. Names of RI-specific AKAPs are highlighted in orange, RII-specific AKAPs are red, and dual-specific AKAPs are black, as indicated. MVB, multi-vesicular bodies; RI/RII, PKA regulatory subunits. Created with BioRender. Smolenski, A. (2026) https://BioRender.com/69jm5is.

Platelet signalling is often initiated at the plasma membrane, and many platelet AKAPs are known to interact with membrane proteins, providing ample opportunities for cAMP/PKA-mediated regulation of signalling events at or close to the plasma membrane (Figure 2). Furthermore, the plasma membrane contains key receptor complexes like the integrin α_IIb_β_3_ and associated proteins required for platelet/platelet interactions. Some plasma membrane AKAPs are known to connect membrane components to the actin cytoskeleton.

#### 5.1.1. Talin-1 (RII)

Talin-1 is a highly abundant platelet protein playing a critical role in integrin activation and platelet aggregation [71,74]. Talin is composed of an N-terminal FERM (4.1, ezrin, radixin, moesin) domain that interacts with integrins in cooperation with kindlin-3 and an extended rod domain composed of 13 bundles of alpha helices, which includes multiple binding sites for the F-actin binding protein vinculin [84].

In the inactive state, the F2 and F3 subdomains of the FERM domain interact with rod domains R9 and R12, preventing membrane and integrin interactions [85]. The binding of active G protein Rap1b to the F0 subdomain has been suggested to trigger talin-1 recruitment to the plasma membrane, followed by integrin α_IIb_β_3_ activation, talin unfolding, and exposure of actin and vinculin binding sites [86]. In platelets, loss of talin-1 led to defective platelet adhesion and aggregation, resulting in prolonged bleeding [74]. A similar phenotype was observed in platelets expressing a talin-1 mutant deficient in Rap1 binding [73]. Surprisingly, disrupting talin-1 autoinhibition by mutation of glutamate 1770 to alanine in the R9 domain resulted in impaired platelet aggregation and in delayed clot retraction, potentially pointing to additional regulatory mechanisms [72].

Recently, Kang et al. reported RII binding of talin-1 [87]. A cryptic RII binding site was mapped to helix 41 of the R10 alpha helical bundle, and RII binding was suggested to require stretch-dependent unfolding of the R9 domain. A potential role for Rap1 triggered talin-1 activation in PKA binding, however, was not addressed. Expression of Talin-1 mutants that do not bind RII led to reduced levels of RII as well as reduced RII serine 99 and VASP serine 157 phosphorylation in focal adhesions of human umbilical vein endothelial cells, as measured by immunofluorescence staining and image analysis. One might speculate that the effect of the above-mentioned glutamate 1770 mutation of talin seen in platelets could be mediated by enhanced recruitment of PKA, supporting platelet inhibition. The proximity between talin-1 and Rap1 could suggest that talin-1-dependent PKA anchoring supports PKA-mediated phosphorylation and regulation of GEFs and GAPs of Rap1, like CalDAG-GEFI and Rap1GAP2 [88,89]. Talin-1 itself was also detected as a possible PKA substrate in platelets by proteomics [59].

#### 5.1.2. Ezrin/Radixin/Moesin (Dual)

The ezrin/radixin/moesin (ERM) family of proteins provides links between plasma membrane-associated proteins and the cortical actin cytoskeleton [90] and each family member is expressed at high levels in platelets. Structurally, ERM proteins are characterised by an N-terminal FERM domain mediating interaction with various plasma membrane-associated proteins, central alpha-helical regions, and a C-terminal actin-binding domain.

Typically, ERM proteins exist in different forms with membrane lipid (phosphatidylinositol (4,5)-bisphosphate) binding and phosphorylation events mediating the transition from inactive to active conformations. Moesin has been shown to interact with the PECAM-1 receptor in platelets [91]. During thrombin-induced platelet activation, moesin is phosphorylated on threonine 558, which enables moesin binding to F-actin [92]. All ERM proteins have been shown to exist in two conformations, an active form that interacts with actin and AKAPs and an inactive one that does not. The transition to the active form depends on phosphorylation of a conserved C-terminal threonine [90].

All three ERM proteins were initially shown to bind RII in gastric cells, and the R binding amphipathic helix was mapped to amino acids 417–432 for ezrin [93]. Later, ERMs were also shown to bind RI in yeast, T-cells, neurons, and hepatocytes [94,95,96]. In airway smooth muscle cells, ezrin was recently shown to enable the phosphorylation of the PKA substrate VASP [97]. A more complex pattern of cAMP signalling regulation by ezrin was reported in colorectal cancer cells. In these cells, PKA RII interacted with threonine 567 phosphorylated ezrin, supporting cell survival. However, displacement of RII from ezrin resulted in RII binding to AKAP1 (also called AKAP149), resulting in apoptosis [98].

The first paper addressing AKAP functions in platelets focused on the ERM protein moesin. Raslan et al. identified moesin as an RI-binding AKAP in platelets [75]. RI and moesin were both shown to localise to lipid rafts of the plasma membrane (Figure 2), and RI’s presence at lipid rafts was stimulated by PGI_2_. RI localisation to lipid rafts was inhibited following treatment of platelets with a poly-arginine-linked membrane-permeable version of the RI-specific disruptor peptide RIAD (RIAD-R11) but not the scrambled control peptide. Loss of RI from platelet lipid rafts resulted in reduced phosphorylation of the membrane-associated PKA substrate protein GPIbβ. Of note, RIAD-R11 treatment also reduced the phosphorylation of a few other unidentified proteins with different molecular weights (75, 100, 165 kDa); however, changes in VASP phosphorylation were not described. RIAD-R11, as well as a stearylated membrane-permeable version of the more general AKAP disruptor peptide Ht31 (St-Ht31), could reverse PGI_2_-induced inhibition of von Willebrand factor (VWF)/ristocetin-induced platelet agglutination. Similar effects were reported for collagen- and thrombin-induced platelet aggregation. Furthermore, RIAD attenuated PGI_2_-mediated inhibition of platelet adhesion to VWF under flow conditions [75]. As RIAD and Ht31 would be expected to interfere with any RI/RII-AKAP interactions, including ezrin and radixin, the aggregation and adhesion experiments do not confirm a specific role for moesin as AKAP in platelets but might highlight the importance of PKA anchoring for cAMP-mediated platelet inhibition in general.

#### 5.1.3. smAKAP (RI)

Two complementary studies have characterised smAKAP (also called AKAP19) as a small membrane-anchored AKAP that binds PKA regulatory subunit RI with nanomolar affinity. The initial research identified smAKAP through cAMP-affinity proteomics in human heart tissue and platelets. This work demonstrated that N-terminal myristoylation and palmitoylation target smAKAP to the plasma membrane. SmAKAP binds PKA-RI with high affinity (Kd 7 nM), leading to colocalization of smAKAP-GFP with PKA-RI at filopodia and cell–cell junctions [76]. In a more recent study, the crystal structure of the smAKAP anchoring domain (AKB peptide) bound to the RIα D/D dimerisation-docking domain was resolved. This analysis revealed a hydrophobic interface with six contact sites that explain RI specificity. Additionally, serine 66 of smAKAP was identified as a PKA-phosphorylation site whose modification disrupts RI binding and destabilises the AKB helix, therefore establishing an auto-inhibitory feedback mechanism for localised cAMP signalling [77]. Despite these mechanistic insights, these results were derived from in vitro assays and lacked in vivo validation, leaving open questions about smAKAP’s physiological function, especially in platelets. Because of its strict membrane location, smAKAP would be predisposed to control PKA functions at the plasma membrane (Figure 2) [51].

#### 5.1.4. PI3Kγ (RII)

The plasma membrane lipid phosphatidylinositol 4,5-bisphosphate (PIP_2_) can be phosphorylated to phosphatidylinositol 3,4,5-trisphosphate (PIP_3_) by phosphoinositide-3-kinases (PI3K). PIP_3_ supports the recruitment of pleckstrin homology (PH) domain-containing proteins like the serine/threonine protein kinase Akt (also known as PKB) to the plasma membrane, initiating various signalling cascades. The catalytic subunit of the gamma isoform of PI3K (PI3Kγ) is an AKAP for RII in cardiomyocytes. The R binding site was mapped to amino acids 126–150, but unexpectedly, this region does not appear to form an alpha helix [99].

Bound PKA was shown to phosphorylate PI3Kγ, leading to reduced kinase activity of PI3Kγ. PI3Kγ was also shown to bind to PDEs 3 and 4. PKA-mediated phosphorylation and activation of these PDEs reduced local cAMP levels and limited PKA actions both in cardiomyocytes [100] and in airway epithelial and smooth muscle cells [101]. The physiological significance of the PI3Kγ/PKA/PDE complex was supported by experiments using membrane-permeable disruptor peptides that blocked PKA binding to PI3Kγ. Loss of PKA binding resulted in loss of PDE activation, leading to elevated cAMP and PKA substrate phosphorylation at the plasma membrane with beneficial effects in models of cystic fibrosis and obstructive airway disease [101].

In platelets, PI3Kγ is known to be activated downstream of GPCRs like the P2Y12 ADP receptor, contributing to Rap1B and integrin α_IIb_β_3_ activation and platelet aggregation [79,102]. Thus, PI3Ks are considered as targets for anti-platelet therapeutics [102,103]. Interestingly, PI3Kγ has been suggested to activate integrin α_IIb_β_3_ through a non-enzymatic mechanism, possibly through its function as a scaffold [78]. It could be hypothesised that, similar to cardiac and airway cells, PI3Kγ-mediated PKA and PDE3 activation could lower local cAMP levels, leading to reduced cAMP/PKA-mediated Rap1B and integrin α_IIb_β_3_ inhibition. However, no changes in whole cell cAMP or phospho-VASP levels were seen in PI3Kγ knockout platelets [78].

#### 5.1.5. Merlin (RI)

Merlin is an ERM-related protein encoded by the *NF2* (Neurofibromin-2) gene. Like ERM proteins, merlin connects plasma membrane structures with the cortical actin cytoskeleton and is thought to be involved in cell contact-dependent regulation of cell proliferation [104]. Merlin is frequently found to be mutated in mesothelioma [105]; however, its role in platelets has not yet been established. Merlin has been described as an RIβ-binding AKAP in neurons [106]. RI is primarily associated with phosphorylated merlin, suggesting that phosphorylation might lead to a conformational change in merlin exposing the R-binding amphipathic helix similar to ERM proteins. The amphipathic helix was mapped to residues 463–480 of merlin, which aligns with other RI binding AKAPs, and RIIα binding was excluded [106].

#### 5.1.6. AKAP5 (RII)

AKAP5 (also AKAP79, H21, or AKAP150 in rodents) supports the formation of signalling complexes at the plasma membrane and facilitates crosstalk between Ca^2+^ and cAMP signalling. The RII binding helix has been identified, and AKAP5 was shown to bind protein kinase C as well as the calcium-dependent protein phosphatase 2B (PP2B, calcineurin). AKAP5 interacts with various membrane receptors, including the β2-adrenergic receptor in cardiomyocytes or the NMDA receptor in neurons [107]. The physiological importance of AKAP5-mediated PKA anchoring for neuronal cell functions was confirmed using a mouse model carrying a deletion of the R-binding helix [108,109]. Importantly, AKAP5-bound PP2B mediated RII dephosphorylation, resulting in enhanced re-capturing of PKA C subunits and thus supporting the termination of C-mediated substrate phosphorylation and cAMP signalling in dendritic spines of neurons [110]. AKAP5 has also been suggested to contribute to PKA-mediated control of Ca^2+^ signalling at the plasma membrane of cardiomyocytes [68,111]. AKAP5 has not been studied in platelets so far.

#### 5.1.7. AKAP12 (RII)

AKAP12 (also AKAP250, Gravin) associates with the plasma membrane, where it is involved in scaffolding multiple signalling pathways, including PKA-RII in the heart and other cells. However, the RII binding helix has not been determined [112]. AKAP12 supports mRNA binding and protein translation at the plasma membrane [113]. Recent studies in airway smooth muscle cells indicate that AKAP12 might be cooperating with ezrin in the regulation of Gs-coupled GPCR signalling [97]. No data on platelet AKAP12 are available so far.

#### 5.1.8. Neurobeachin (RII)

Neurobeachin (also called Lysosomal-trafficking regulator 2, or Protein BCL8B) has been described as a neuronal protein involved in membrane receptor targeting and synaptic function [114]. A RII binding site has been defined, and PKA binding has been shown to facilitate phosphorylation and internalisation of synaptic membrane receptors [115]. Interestingly, neurobeachin has been studied in the context of autism in platelets, as platelets are considered a model for the study of neuronal disorders due to the expression of similar proteins in neurons and platelets. Platelets from heterozygous neurobeachin knockout mice (Nbea+/−) exhibited slightly smaller dense granules, reduced processing of talin-1, and altered protein phosphorylation patterns, compared to wild-type platelets [80]. The impact of reduced neurobeachin levels on cAMP/PKA signalling was not addressed in this study.

In conclusion, a broad range of AKAPs might be targeting cAMP/PKA to the plasma membrane, although the precise function of individual AKAPs and the coordination of membrane AKAP compartments still need to be determined (Figure 2). The ERM protein moesin has been confirmed as a plasma membrane RI binding AKAP in platelets, and moesin was shown to be required for phosphorylation of GPIbβ in platelets. Ezrin might be involved in the phosphorylation of VASP based on data from other cells. Talin-1 and ERM proteins appear to be expressed at particularly high levels in platelets compared to other AKAPs. Nevertheless, it is not likely that platelet PKAs would be linked preferentially to talin-1 and ERMs due to their conditional nature of R binding: talin-1 requires stretch exposure, whereas ERMs must be phosphorylated to permit R binding. Thus, talin-1 and ERMs might facilitate PKA I and II anchoring during specific states of platelet function. SmAKAP, on the other hand, might enable a more constitutive PKA I compartment at the plasma membrane due to its permanent lipid-mediated membrane binding. Based on studies in other cells, AKAP5 could be involved in PKA-mediated control of platelet Ca^2+^ signalling at the plasma membrane.

### 5.2. Endosomal AKAPs

A few AKAPs found to localise to endosomal compartments in other cells are also expressed in platelets (Figure 2). Some of these endosomal AKAPs might be involved in receptor endocytosis and recycling, in the regulation of granule development and release, or in other membrane sorting events in platelets. Recruitment of cAMP/PKA to these sites could provide novel control mechanisms.

#### 5.2.1. Rab32 (RII)

Rab32 is a small G protein that uniquely doubles as an A-kinase anchoring protein, tethering PKA-RII to distinct organelles and directing localised phosphorylation events. The first evidence of Rab32′s AKAP activity came from a yeast-two-hybrid screen that mapped an amphipathic helix (amino acids 178–197) as the core PKA-anchoring motif [116]. In HEK-293 cells, wild-type Rab32 co-precipitated with endogenous PKA and enriched PKA activity at mitochondria [116]. Within mitochondria-associated membranes, Rab32 anchored PKA phosphorylated substrates like the pro-apoptotic protein Bad and the mitochondrial fission GTPase Drp1. These effects are associated with delayed apoptosis [117]. Rab32′s AKAP function is essential for maintaining Golgi structure and promoting cell migration through PKA-mediated phosphorylation of optineurin, a protein also expressed in platelets [118], while its lysosomal association is necessary for promoting cell growth and metabolism by facilitating mTORC1 recruitment and activity [119].

While Rab32 has been established as an AKAP that recruits PKA signalling to the mitochondria-associated membrane, lysosomes and the Golgi apparatus in various cell types, its specific role or function as an AKAP within platelets remains largely undefined. Research closest to the platelet lineage used a human megakaryocytic cell line (MEG-01) to model dense granule biogenesis. This model showed that Rab32, along with Rab38, was a crucial regulator in the late endocytic pathway, promoting the fusion of cargo-containing vesicles with multivesicular bodies/immature dense granules [120]. Rab32 localization to the limiting membrane of immature dense granules indicated its role in regulating the biosynthetic transport pathway required for the maturation of these platelet-specific organelles. However, these specific studies focused on the membrane trafficking functions of Rab32 in megakaryocytes and did not investigate its parallel activity as an AKAP in the context of dense granule formation or in fully differentiated platelets.

#### 5.2.2. AKAP10 (Dual)

AKAP10 (also called dual specificity A kinase-anchoring protein 2, D-AKAP2) was identified in yeast two-hybrid screening experiments as RI and RII binding AKAP [121]. The crystal structure of the C-terminal R binding helix of AKAP10 (amino acids 631–649) bound to RII has been analysed in detail [63]. The very C-terminus of AKAP10 contains a PDZ binding motif which interacts with PDZ domains of PDZK1, NHERF1 and NHERF2, and it has been shown that AKAP10 can interact with both RII and PDZ domains simultaneously [122]. In addition, AKAP10 contains two tandem regulator of G-protein signalling (RGS) domains which interact with GTP-bound small G proteins Rab11 and Rab4 [123]. AKAP10 co-localised with endosomes and promoted the accumulation of recycling proteins in the Rab4/Rab11-positive endocytic recycling compartment in mammalian cells. Interestingly, the interaction of AKAP10 with Rab4/Rab11 endosomes was also detected in Drosophila and the Drosophila Pkaap, an orthologue of AKAP10, stimulated Rab11-dependent vesicle traffic [124]. AKAP10 has also been suggested to localise to the outer mitochondrial membrane, supporting compartmentalisation of cAMP in airway smooth muscle cells [125]. AKAP10 was detected in a cAMP-binding proteome study in platelets [70]; however, no functional data on platelet AKAP10 is available yet. Platelets express some of the established AKAP10 binding partners like Rab4, Rab11, and NHERF1 [43,50] and Rab4 has been suggested to regulate alpha granule secretion [126].

#### 5.2.3. AKAP11 (Dual)

AKAP11 (also known as AKAP220) was detected following cAMP pull-down in platelets [70]. AKAP11 mutations have been associated with schizophrenia and bipolar disorder, and AKAP11 was recently shown to play a role in autophagy via RI binding [127]. Previously, AKAP11 was identified as an RII binding protein that also interacts with IQGAP1 and regulates cell migration [128].

Taken together, platelets express Rab32 and AKAP10 and 11 as AKAPs that might link PKA signalling to endosomal pathways and to granule biogenesis and release. Rab32 is an RII-specific AKAP expressed at high levels in platelets, which plays a confirmed role in dense granule biogenesis, at least in megakaryocytes. AKAPs 10 and 11 can bind both RI and RII. Links between AKAP10 and Rab4 might indicate a role for AKAP10 in alpha granule release.

### 5.3. Golgi, Mitochondria, and DTS-Linked AKAPs

Proteomics indicate expression of at least five AKAPs that are likely to be associated with organelles like the Golgi, mitochondria, or the DTS in platelets (Figure 2).

#### 5.3.1. ACBD3 (Dual)

ACBD3 (Acyl-CoA-Binding Domain-Containing 3, also called Golgi resident protein GCP60 or peripheral-type benzodiazepine receptor-associated protein PAP7) is involved in the synthesis of steroids and sphingolipids and in membrane trafficking, possibly through binding to the outer mitochondrial membrane or through supporting the integrity of the Golgi apparatus. ACBD3 contains a C-terminal acyl-CoA-binding domain and an N-terminal GOLD (Golgi Dynamics) domain and has been shown to interact with numerous proteins, including SNARE proteins and phosphatidylinositol 4-kinase beta, which are also present in platelets [129,130]. ACBD3 was initially identified as RI AKAP by yeast two-hybrid screening and RI binding was confirmed by GST-pulldown assay; however, the RI binding amphipathic helix was not defined [131]. Evidence for a potential role for ACBD3 in the formation of a mitochondrial PKA compartment was provided by Sherpa et al., who showed co-localisation of RI and RII with ACBD3 at mitochondria of rat ventricular myocytes [132]. However, no functional data on PKA anchoring were presented in this study. ACBD3 has not been studied in platelets.

#### 5.3.2. BIG2 (Dual)

BIG2 (Brefeldin A-inhibited guanine nucleotide-exchange protein 2) is a guanine-nucleotide exchange factor required for the activation of small G proteins of the Arf family involved in vesicular transport. BIG2 localises to the trans-Golgi network (TGN) and BIG2-mediated ARF activation, and regulates the recruitment of adaptor protein 1 (AP-1) and GGA1 to the TGN to facilitate the formation of transport vesicles trafficking between the TGN and endosomes [133]. BIG2 was shown to bind RI and RII in yeast-two hybrid experiments [134]. An interaction between BIG2 and RI was confirmed by co-immunoprecipitation of over-expressed proteins as well as at endogenous protein levels in HepG2 cells. Three amphipathic helices were identified as potential R binding sites: (A) amino acids 34–48, (B) 284–298, and (C) 525–539. RIIβ on the other hand was shown to regulate BIG2-mediated traffic of exosome-like vesicles containing type I tumour necrosis factor (TNF) receptor to the plasma membrane of human umbilical vein endothelial cells [135]. In this study, long-term activation of PKA for 24 h was shown to facilitate TNF transport and mutation of critical residues in R binding sites B and C resulted in a loss of BIG2 effects on TNF transport. These data suggest that RII-mediated anchoring of PKA to BIG2 might stimulate vesicle transport. Interestingly, BIG2 was found to interact with PDE3 in HeLa cells [136] and PKA-mediated phosphorylation of BIG2 has been reported to reduce the GEF activity of BIG2 [137]. Recent studies indicate that BIG2 might also be linked functionally to RhoA pathways in neurons [138]. BIG2 has not been studied in platelets to date.

#### 5.3.3. AKAP7 (RII)

AKAP7 (also called AKAP18) is a PKA-RII-specific anchoring protein that is expressed in various short and long isoforms. Evolutionary analysis showed that AKAP7 long-form splice variants (γ and δ) have undergone rapid change and contain species-specific mutations, in contrast to the highly conserved short forms alpha and beta (ɑ and β) [139]. Human genomic sequencing identified a nucleotide insertion event in AKAP7δ that caused a shift in the reading frame and created a downstream start codon, thereby favouring the translation of AKAP7γ isoform and reducing the production of functional AKAP7δ. Therefore, this data suggested that in humans, AKAP7γ is the predominantly long-form isoform, whereas AKAP7δ may have limited physiological relevance [139]. The short isoforms localise to membrane-bound ion-channels, which anchor PKA to L-type Ca^2+^ channels and ENac to regulate cardiac and renal ion transport. This regulates channel phosphorylation and activity in the heart, brain, kidney and lung [139]. AKAP7 did not appear to be involved in PKA-mediated Ca^2+^ regulation in mouse cardiomyocytes [140]. In contrast, a related study suggested a role for AKAP7γ/δ in the control of Ca^2+^ at the sarcomere of cardiomyocytes [141].

AKAP7γ and δ are expressed in human platelets and bind the type-II regulatory subunit of PKA, which was confirmed by cAMP pull-down and proximity-ligation assays. AKAP7δ formed a cytosolic complex with PDE3A/PKA-RII in platelets (Figure 2). This complex anchored activated PKA and mediated prostacyclin-induced PDE3A phosphorylation. Disruption of this complex reduced PDE3A activation and raised basal cAMP levels, which demonstrates a feedback loop that limits platelet activation. Co-immunoprecipitation studies revealed the association of PDE3A with PKA RII and AKAP7δ but not AKAP7γ [83]. These studies demonstrate a functional role for AKAP7δ in humans, which contradicts the prior evolutionary work that argues that AKAP7δ is less functional than AKAP7γ. A dimerisation study provided mechanistic detail on AKAP7γ in a kidney-derived cell line. AKAP7γ can form both homodimers and heterodimers with other long isoforms (AKAP7δ), and this oligomerization creates higher-order scaffolds that concentrate PKA near its substrates, thereby enhancing phosphorylation efficiency by 1.91-fold compared to a monomeric state according to computational modelling [142].

#### 5.3.4. AKAP1 (Dual)

AKAP1 (also named Dual specificity A-kinase-anchoring protein 1, D-AKAP1, or S-AKAP84, or AKAP 149) was originally identified as the RII binding protein [143]. AKAP1 was later found to bind both RI and RII in the yeast two-hybrid system [144]. AKAP1 localises to the outer membrane of mitochondria and the endoplasmic reticulum [145,146]. However, in adipocytes, co-localisation of endogenous AKAP1 and RII could not be confirmed [147]. Endogenous AKAP1 was also shown to interact with GEF-H1 (ARHGEF2, Lfc), a GEF of RhoA and a known PKA substrate in platelets [54,148]. Another role of AKAP1 appears to be the anchoring of mRNA and the control of protein translation at the mitochondrial membrane [113].

#### 5.3.5. AKAP9 (RII)

AKAP9 (also AKAP350, AKAP450, or Centrosome- and Golgi-localised PKN-associated protein CG-NAP) is a large anchoring protein that binds specifically to type II PKA, the cAMP-regulated exchange factor Epac1, and phosphodiesterase PDE4D3 and organises these components into localised signalling units. AKAP9 also associates with microtubules and microtubule-regulatory complexes, including the γ-tubulin ring complex, dynein, EB1 and Golgi proteins such as GM130 and CDK5RAP2, which promote microtubule nucleation and polymerisation [149]. In endothelial cells, AKAP9 co-localised with GM130, bound Epac1, and was essential for Epac1-induced barrier strengthening by accelerating microtubule growth and supporting integrin-mediated adhesion, while basal permeability, Rap1 activation, and PKA-mediated barrier responses remained intact [150]. In a recent study on non-small cell lung cancer cells, AKAP9 was shown to form a ternary Golgi-anchored complex with PDE4DIP and PKA-RII, stabilising the regulatory subunit and supporting tumour growth. Silencing AKAP9 destabilised both PDE4DIP and PKA-RII, which disrupted Golgi anchoring and enhanced RII ubiquitination [149,151]. AKAP9 has not yet been characterised in platelets.

In summary, platelets express Golgi, mitochondria, and DTS-associated AKAPs ACBD3, BIG2, AKAP7δ, AKAP1 and AKAP9 (Figure 2). Golgi-associated AKAPs might have functional roles in granule biogenesis in platelets. AKAP7δ is one of the few AKAPs that have been studied in platelets. Based on studies in cardiomyocytes, AKAP7δ would be predicted to contribute to PKA-mediated regulation of Ca^2+^-release from the DTS; however, this has not been investigated in platelets so far. AKAP9 might also connect PKA to microtubules (see MAP2 as another microtubule-associated AKAP below).

### 5.4. AKAPs Linked to the Actin Cytoskeleton

Some of the plasma membrane AKAPs mentioned above are also linked to the actin cytoskeleton. In addition, platelets express several other actin-binding AKAPs which could contribute to cAMP/PKA-mediated control of platelet adhesion, aggregation, and clot retraction.

#### 5.4.1. WAVE1 (RII)

WAVE1 (Wiskott-Aldrich syndrome protein family member 1) is a member of the WASP/Scar (WAVE) family of adaptor proteins that links G proteins Rac1 and Arf1 to the Arp2/3 complex which promotes actin nucleation and branching required for lamellipodia formation [152,153]. WAVE1 acts as an AKAP that binds to PKA RII and Abl tyrosine kinase, assembling a scaffold that can be recruited to focal adhesions and sites of cytoskeletal remodelling [154]. The protein contains a central region that mediates PKA-RII interactions (amino acids 493–510) and overlaps with an actin-binding (VPH) motif, making PKA and actin binding mutually exclusive in vitro. However, PDGF stimulation drives a coordinated translocation of WAVE-1, PKA and Abl to lamellipodia and actin “ring” structures in fibroblasts, indicating that distinct cellular pools can co-exist at sites of actin remodelling [154]. Macrophage studies reveal that oxidised phospholipids trigger WAVE-1-mediated actin spreading in a PKA-dependent manner [155]. WAVE2 has also been suggested to act as an AKAP for RII; however, no detailed studies have been performed on the AKAP activity of WAVE2 so far [65].

Human platelets express all three WAVE isoforms (WAVE1, WAVE2, WAVE3) [81]. WAVE1 has been detected in platelet proteomic studies [43,70]; however, PKA anchoring by WAVE1 has not been analysed in platelets. In aggregating TRAP-stimulated platelets, WAVE-1 was recruited into the polymerized actin cytoskeleton in an integrin-dependent manner, supporting its role in actin remodelling during platelet aggregation [81]. Fluorescence microscopy in human platelets showed that WAVE1 does not localise exclusively to the lamellipodial tip, but it had a more scattered distribution compared to WAVE2. WAVE1 may scaffold PKA/Abl at actin-nucleation sites or early adhesions [81]. Importantly, WAVE1 was shown to be essential for GPVI-mediated actin assembly and aggregation in mouse platelets but was not required for GPCR-driven responses and shear-dependent thrombus formation. This receptor-specific defect suggests that WAVE-1 couples GPVI tyrosine kinase signalling to localised actin polymerization [82].

#### 5.4.2. AKAP-Lbc (RII)

AKAP-Lbc (also AKAP13) is a dual-function RII-specific AKAP that was first identified when extension of the canonical Ht31 fragment revealed that this sequence is embedded within a much larger splice variant of the Lbc oncogene. Cloning yielded a full-length cDNA encoding a 2817 amino acid protein of approximately 312 kDa [156]. This large scaffolding protein contains two ankyrin repeats (amino acids 166–224), a type-II PKA binding domain (1236–1257), a C1-homology region (1792–1830, cysteine-rich motif homologous to CI of PKC), the DH domain (1998–2190), and the PH domain (2232–2335) that is characteristic of Rho family GEFs [157]. A 257 amino acid fragment near the C terminus of AKAP-Lbc interacts with constitutively active Gα_12_ (Q229L) but shows no significant affinity for Gα_13_ [158]. The catalytic activity of AKAP-Lbc is tightly regulated by a negative feedback loop involving anchored PKA-mediated phosphorylation of AKAP-Lbc at serine 1565, leading to 14-3-3 binding. In cellular models, the elevation of intracellular cAMP via forskolin treatment resulted in an increase in the co-immunoprecipitation of 14-3-3 with AKAP-Lbc, an effect that was sensitive to the specific PKA inhibitor PKI. The PKA-dependent recruitment of 14-3-3 serves as a potent inhibitory signal for AKAP-Lbc’s Rho-GEF activity. In migrating cells, AKAP-Lbc’s PKA anchoring function might be more important than its GEF activity. Using FRET-based PKA imaging, AKAP-Lbc was identified as a major contributor to polarised PKA activity gradients at the leading edge. Disruption of PKA anchoring with the stHt31 peptide completely abolished gradients, demonstrating that anchored PKA, not freely diffusible catalytic subunits, generates localised signalling.

AKAP-Lbc is often referred to as functioning in a bidirectional manner wherein G_α12_ stimulates RhoA through AKAP-Lbc, while PKA/14-3-3 binding inhibits this. Despite the importance of G_α12/13_-Rho signalling and cAMP-PKA pathways in platelet activation, shape change, and thrombus formation, AKAP-Lbc has not been examined in either megakaryocytes or platelets, leaving its potential role in haemostasis or thrombosis unexplored.

#### 5.4.3. AKAP2 (Dual)

AKAP2 (also called Paralemmin-2-AKAP2, or AKAP-KL) is an actin-binding protein that also interacts with extracellular signal-regulated kinase 1 [159]. AKAP2 supports the regulation of aquaporin channels by binding and enabling aquaporin phosphorylation by PKA [160]. The R binding helix of AKAP2 has not been mapped. A possible dual specificity of AKAP2 for RI and RII was suggested in a study comparing efficiencies of various cAMP beads to pull down Rs and AKAPs [161]. AKAP2 has been detected in cAMP pull-down experiments in human platelets. Of note, treatment of platelets with collagen-related peptide, an agonist of the GPVI receptor, increased binding of AKAP2 to cAMP-beads [70].

In conclusion, WAVE1 and AKAP-Lbc are prominent actin-linked AKAPs expressed in platelets which might provide platforms for cAMP/PKA-mediated control of actin-binding proteins like VASP, as well as of GEFs and GAPs of G proteins Rac1 and RhoA which mediate actin remodelling required for lamellipodia formation, adhesion, aggregation, and clot retraction.

### 5.5. AKAPs Linked to Microtubules and Intermediate Filaments

Three AKAPs known to interact with microtubules or intermediate filaments have been detected in platelets, albeit at low copy number levels (Figure 2). Preliminary data suggest that the core microtubule proteins alpha and beta tubulin might act as AKAPs for RI [162]. However, the specific tubulin subtypes have not been defined, and the interaction sites between tubulins and RI have not been determined.

#### 5.5.1. MAP2 (RII)

Microtubule-associated proteins (MAPs) regulate the stability and dynamics of microtubules [163]. MAP2 was the first AKAP ever to be described [164,165] and its importance for PKA localisation was confirmed in various types of neurons [166]. Interestingly, MAP2 binding of PKA determined localisation of inactive PKA in dendrites, whereas PKA activation led to translocation of PKA C subunits into dendritic spines, leading to substrate phosphorylation. MAP2 has not been studied in platelets. Gene ontology analysis of potential PKA substrates found in platelets by phospho-proteomics indicates that 37 of the 222 identified substrates might be linked to microtubules [59,167].

#### 5.5.2. Pericentrin (RII)

Pericentrin is a centrosomal protein that is associated with tubulin and multiple other proteins [168]. Pericentrin was identified as RII-specific AKAP lacking an AKB helix. Instead, a unique 100 amino acid sequence was shown to be required for R binding [169]. Interestingly, pericentrin was also shown to interact with AKAP9; however, the exact function of pericentrin is not clear [170].

#### 5.5.3. Synemin (RII)

Synemin is a multifunctional adapter protein described to attach to the intermediate filament protein vimentin, which is also expressed in platelets, as well as to protein phosphatase 2A, providing a link to Akt signalling [171]. The RII binding helix has been defined, and colocalisation of synemin, RII and desmin in Z-lines of cardiomyocytes has been shown. The functional significance of PKA anchoring by synemin is not clear [172].

Little data is available regarding PKA regulation of microtubules or intermediate filaments in platelets. Microtubules play an essential role in megakaryopoiesis and proplatelet formation and cAMP/PKA signalling was shown to promote megakaryopoiesis, but to suppress platelet production [173,174]. MAP2 and pericentrin might link microtuble associated proteins to the cAMP signalling system. The RhoGEF GEF-H1 localises to microtubules and was shown to be phosphorylated by PKA in platelets. However, PKA mediated phosphorylation of GEF-H1 was not affected by microtubule depolymerization indicating that GEF-H1 is not phosphorylated at microtubules [54]. The actin linked AKAP and RhoGEF AKAP-Lbc might bind to microtubules as well [175].

### 5.6. Cytosolic AKAPs

#### 5.6.1. RSK1 (RI)

The serine/threonine protein kinase RSK1 (Ribosomal protein S6 kinase alpha-1) is a noncanonical RI specific AKAP which is also linked to AKAP1 [176]. As RSK1 is part of the Ras/mitogen-activated protein kinase (MAPK) pathway and a substrate of extracellular signal-regulated kinases 1 and 2 (ERK1/2) [177]. Although RSK1 has not been studied in platelets, the phosphoinositide-dependent protein kinase 1 (PDK1), another kinase upstream of RSK1, plays an important role in PI3K dependent platelet activation [178]. Furthermore, crosstalk between cAMP and MAPK pathways has been demonstrated in platelets [179].

#### 5.6.2. Neurochondrin (RII)

Neurochondrin has been described as neuronal protein involved in ERK signalling and action potential regulation [180]. Neurochondrin was identified as RII binding AKAP in pull-down assays using cAMP agarose beads and brain tissue [181]. Two high-affinity sites (in the low nM range) were characterised to mediate RII binding, but these did not match the classical AKB helix sequences. The functional role of PKA binding is unknown, and neurochondrin has not been studied in platelets to date.

## 6. Conclusions and Future Directions

Platelets express many AKAPs with a wide range of potential subcellular distributions reflecting the complex spectrum of PKA substrates. Only two of these, the plasma membrane-associated AKAP moesin and the possibly DTS linked AKAP7, have been characterised in detail. Other platelet AKAPs are likely localised at the OCS, the Golgi, mitochondria, endosomes, MVB, alpha and dense granules, and the cytoskeleton (Figure 2). AKAPs are expected to provide critical guidance to PGI_2_/cAMP/PKA mediated substrate phosphorylation leading to coordinated inhibition of platelet functions.

Despite recent advances in cAMP/PKA and AKAP biology, current understanding of AKAP dependent signalling in platelets remains limited, and many open questions remain to be addressed.

1. What is the exact identity, expression level, R specificity, and subcellular localisation of platelet AKAPs?

While proteomic and transcriptomic analyses suggest potential AKAPs in platelets, their precise expression level and subcellular location remain unclear. Detailed quantitative studies, including evaluation of R affinities at endogenous protein levels, and high-resolution imaging studies using specific antibodies are required to improve current understanding [42,182].

2. How are AKAP-directed subcellular compartments linked to PKA-mediated substrate phosphorylation and platelet inhibition?

Given the limited spatial range of C subunits following release from the PKA holoenzyme, specific subsets of PKA substrates are likely to be linked to specific AKAPs. For example, PKA-mediated control of Ca^2+^-release from intracellular stores might involve substrates like RGS18 or the IP_3_ receptor but the AKAP(s) targeting PKA to these substrates are not known. Cell models like induced pluripotent stem cell-derived megakaryocytes and platelets expressing AKB-deficient AKAPs could be used to address these questions [183].

3. What is the role of dynamic PKA redistribution between different AKAPs?

In some cells, dynamic redistribution of PKAs between AKAPs has been observed. Changes in PKA localisation could be relevant during different stages of platelet activation or in subsets of platelets within developing thrombi [182].

4. How are PDEs integrated into AKAP functions in platelets?

PDEs are critical regulators of cAMP signalling in many cells, and links between AKAP7δ and PDE3 have been described in platelets. It would be interesting to evaluate if PDE3 can also link to other AKAPs, and to identify any possible connections between platelet AKAPs and PDEs 2 and 5. Specific PDE inhibitors could help address these questions [36,184].

5. What is the role of RI phase separation for the compartmentalisation of cAMP signalling in platelets?

Liquid–liquid phase separation has been suggested to contribute to the lowering of cytosolic cAMP levels through the formation of RIα and RIβ bodies harbouring cAMP and PKA. Similar RI bodies might be present in platelets which could be studied using megakaryocyte and platelet models expressing labelled RI [47].

6. Are AKAPs involved in the coordination of cAMP/PKA and cGMP/PKG pathways?

Both cyclic nucleotide pathways are known to be closely connected in platelets, resulting in the phosphorylation and regulation of many identical substrate proteins. AKAPs (and GKAPs) might provide critical platforms enabling signalling coordination. To address this point, careful dissection of cyclic nucleotide pathways in platelets using specific inhibitors would be required [185].

7. How are AKAPs integrated into platelet-activating signalling pathways?

Platelet activation pathways are known to be closely intertwined with cAMP-dependent inhibitory pathways, enabling precise control of platelet responses. AKAPs could play an important role in facilitating these interactions. Detailed analysis of signalling pathways in AKAP knockout models might help to address this point.

8. What is the role of AKAPs in the increased PGI_2_ sensitivity of platelets in response to physical exercise?

Exercise training has been associated with improved PGI_2_/cAMP/PKA responses, leading to enhanced platelet inhibition, which could contribute to the anti-thrombotic effects of exercise [186,187]. The mechanisms underlying this effect are unclear, and changes in IP receptor or PDE3 expression have been ruled out [187,188]. Changes in AKAP expression or function could contribute to the beneficial effects of exercise.

Studies of AKAP functions in general and in platelets in particular are facing a couple of challenges. Since AKAPs are often multi-domain scaffolding proteins, investigating the specific role of PKA binding can be difficult. Furthermore, defining subcellular compartments will be experimentally demanding, since platelets are small cells undergoing a multitude of dynamic spatial and temporal changes during activation, aggregation, and retraction. In addition, different platelet subpopulations might be using AKAPs in specific ways [189].

Improved understanding of AKAP functions has the potential to open new avenues for anti-platelet therapies. Interfering with or stabilising AKAP/R interactions could be beneficial depending on the context. Peptides mimicking the amphipathic R binding helix of AKAPs like RIAD-R11 or St-Ht31 have been used successfully to address research questions. However, the mechanisms involved in the entry and subcellular distribution of cell-permeable peptides require further study [190]. Alternative approaches to eliminate specific AKAPs could involve proteolysis-targeted chimeras [191]. On the other hand, it might be of value to stabilise specific AKAP/R complexes using small molecules in order to support inhibitory cAMP signalling and thus to prevent thrombus formation. This ‘molecular glue’ approach has been successfully applied in the context of protein degraders [192,193].

## Figures and Tables

**Table 1:** A-kinase anchoring proteins identified in human platelets: expression, localisation, and PKA specificity. This table summarises AKAPs for which evidence of expression in platelets has been reported. For each AKAP, gene and protein names, UniProtKb accession numbers and detection in platelet transcriptomic and proteomic datasets are indicated. Copy numbers per platelet are shown where available based on quantitative proteomics [43]. “No copy” indicates that the protein was detected, but no copy number value was obtained. Presence in cAMP-affinity enrichment experiments using cAMP-agarose beads (cAMP beads) [70] is indicated as evidence of functional PKA interaction. PKA regulatory subunit specificity (RI, RII or both), reported or predicted subcellular location, and key platelet-relevant references are listed. “v” denotes that the protein was present in a validation proteome [43]. ✔ indicates the presence of the AKAP in the platelet proteome, RNA or cAMP bead pull-down.

Gene Name	Protein Name(s)	UniProtKb	Platelet RNA	Platelet Proteome	cAMP Beads	Copy Number	PKA-R Specificity	Subcellular Location	Platelet Papers
**Plasma membrane AKAPs**									
*TLN1*	Talin-1	Q9Y490	✔	✔	✔	115,816	RII	Plasma membrane	[71,72,73,74]
*MSN*	Moesin	P26038	✔	✔	✔	34,798	Dual	Plasma membrane	[75]
*RDX*	Radixin	P35241	✔	✔	-	15,522	Dual	Plasma membrane	
*EZR*	Ezrin	P16311	✔	✔	-	13,326	Dual	Plasma membrane	
*C2orf88*	smAKAP, AKAP19	Q9BSF0	✔	✔	-	5140	RI	Plasma membrane	[76,77]
*PIK3CG*	PI3Kγ	P48736	✔	✔	-	1254	RII	Plasma membrane	[78,79]
*NF2*	Merlin	P35240	✔	✔	-	934	RI	Plasma membrane	
*AKAP5*	AKAP5, AKAP79, H21	P24588	✔	✔	-	No copy	RII	Plasma membrane	
*AKAP12*	AKAP12, AKAP250, Gravin	Q02952	✔	✔	-	No copy	RII	Plasma membrane	
*NBEA*	Neurobeachin	Q8NFP9	✔	✔	-	v	RII	Plasma membrane	[80]
**Endosomal AKAPs**									
*RAB32*	Rab32	Q13637	✔	✔	-	8860	RII	Dense granules, Endosomes	[74]
*AKAP10*	AKAP10, D-AKAP2	O43572	✔	✔	✔	819	Dual	Endosomes, Mitochondria	
*AKAP11*	AKAP11, AKAP220	Q9UKA4	✔	-	✔	No copy	Dual	Endosomes	
**Golgi, Mitochondria, and DTS-linked AKAPs**									
*ACBD3*	ACBD3, GCP60, PAP7	Q9H3P7	✔	✔	-	1586	Dual	Golgi, Mitochondria	
*ARFGEF2*	BIG2	Q9Y6D5	✔	✔	-	1003	Dual	Golgi, Endosomes	
*AKAP7*	AKAP 7, AKAP18	O43687	✔	✔	✔	v	RII	DTS	[72]
*AKAP1*	AKAP1, D-AKAP1, S-AKAP84, AKAP149	Q92667	✔	✔	✔	v	Dual	Mitochondria	
*AKAP9*	AKAP9, AKAP350, AKAP450, CG-NAP	Q99996	✔	✔	✔	v	RII	Golgi, Centrosome	
**Actin-binding AKAPs**									
*WASF1*	WAVE-1	Q92558	✔	✔	-	1353	RII	Cytoskeleton	[81,82]
*AKAP13*	AKAP-Lbc, AKAP13	Q12802	✔	✔	-	612	RII	Cytoskeleton	
*PALM2AKAP2*	AKAP2, Paralemmin-2-AKAP2, AKAP-KL	Q9Y2D5	✔	✔	✔	No Copy	Dual	Cytoskeleton	
**Microtubule and intermediate filament binding AKAPs**									
*MAP2*	MAP2	P11137	✔	✔	✔	No copy	RII	Microtubules	
*SYNM*	Synemin	O15061	✔	✔	-	No copy	RII	Intermediate filaments	
*PCNT*	Pericentrin, Kendrin	O95613	✔	✔	-	v	RII	Microtubules	
**Cytosolic AKAPs**									
*RPS6KA1*	RSK1, MAPKAPK-1a	Q15418	✔	✔	-	1320	RI	Cytosol	
*NCDN*	Neurochondrin	Q9UBB6	✔	✔	-	v	RII	Cytosol	

## Data Availability

No new data were created or analyzed in this study.

## Abbreviations

The following abbreviations are used in this manuscript:

AC	Adenylyl cyclase
ADP	Adenosine diphosphate
AKAP	A-kinase anchoring protein
AKAR1	A-kinase reporter 1
AKB	A-kinase binding domain
C	Catalytic subunit of protein kinase A
Ca^2+^	Calcium
cAMP	Cyclic adenosine monophosphate
D/D	Docking dimerization domain
DAG	Diacylglycerol
DTS	Dense tubular system
ERM	Ezrin/radixin/moesin
FRET	Förster resonance energy transfer
GAP	GTPase-activating protein
GPCR	G-protein coupled receptor
IP_3_	Inositol 1,4,5-triphosphate
ITIM	Immunoreceptor tyrosine-based inhibition motif
MAM	Mitochondria-associated membranes
MVB	Multivesicular body
NF2	Neuorfibromin-2
NO	Nitric oxide
OCS	Open canicular system
OPTN	Optineurin
PAR	Protease-activated receptor
PDE	Phosphodiesterase
PECAM-1	Platelet endothelial cell adhesion molecule 1
PGI_2_	Prostacyclin
PH	Pleckstrin homology
PIP_2_	Phosphatidylinositol, 4,5-biphosphate
PIP_3_	Phosphatidylinositol 3,4,5-triphosphate
PKA	Protein kinase A
PKC	Protein kinase C
PKG	Protein kinase G
PP2B	Protein phosphatase 2B
R	Regulatory subunit of protein kinase A
RAIN	Receptor associated independent cAMP nanodomains
RGS	Regulator of G-protein signalling
TGN	Trans-Golgi network
TNF	Tumour necrosis factor
TxA_2_	Thromboxane A2
TRAP	Thrombin receptor activating peptide
VASP	Vasodilator-stimulated phosphoprotein
VWF	Von Willebrand Factor

## References

[1] Gremmel T., Frelinger A., Michelson A. (2016). Platelet Physiology. Semin. Thromb. Hemost..

[2] Smolenski A. (2012). Novel roles of cAMP/cGMP-dependent signaling in platelets. J. Thromb. Haemost..

[3] Bye A.P., Unsworth A.J., Gibbins J.M. (2016). Platelet signaling: A complex interplay between inhibitory and activatory networks. J. Thromb. Haemost..

[4] Stefanini L., Bergmeier W. (2018). Negative regulators of platelet activation and adhesion. J. Thromb. Haemost..

[5] Raslan Z., Aburima A., Naseem K.M. (2015). The Spatiotemporal Regulation of cAMP Signaling in Blood Platelets—Old Friends and New Players. Front. Pharmacol..

[6] Yadav R., Zaccolo M. (2025). GPCR signaling via cAMP nanodomains. Biochem. J..

[7] Bock A., Irannejad R., Scott J.D. (2024). cAMP signaling: A remarkably regional affair. Trends Biochem. Sci..

[8] Gresele P., Momi S. (2022). Novel approaches to antiplatelet therapy. Biochem. Pharmacol..

[9] Mackman N., Bergmeier W., Stouffer G.A., Weitz J.I., Mackman N., Bergmeier W., Stouffer G.A., Weitz J.I. (2020). Therapeutic strategies for thrombosis: New targets and approaches. Nat. Rev. Drug Discov..

[10] Thon J.N., Italiano J.E. (2012). Platelets: Production, Morphology and Ultrastructure. Handbook of Experimental Pharmacology.

[11] Brewer D. (2006). Max Schultze (1865), G. Bizzozero (1882) and the discovery of the platelet. Br. J. Haematol..

[12] Ribatti D., Crivellato E. (2007). Giulio Bizzozero and the discovery of platelets. Leuk. Res..

[13] Selvadurai M., Hamilton J. (2018). Structure and function of the open canalicular system—The platelet’s specialized internal membrane network. Platelets.

[14] Banerjee M., Whiteheart S.W. (2017). The Ins and Outs of Endocytic Trafficking in Platelet Functions. Curr. Opin. Hematol..

[15] Yao H.H.Y., Kahr W.H.A. (2025). Molecular basis of platelet granule defects. J. Thromb. Haemost..

[16] Rosado J.A. (2011). Acidic Ca^2+^ stores in platelets. Cell Calcium.

[17] Gay L., Felding-Habermann B. (2011). Contribution of platelets to tumour metastasis. Nat. Rev. Cancer.

[18] Engelmann B., Massberg S. (2013). Thrombosis as an intravascular effector of innate immunity. Nat. Rev. Immunol..

[19] Suzuki-Inoue K., Tsukiji N. (2024). A role of platelet C-type lectin-like receptor-2 and its ligand podoplanin in vascular biology. Curr. Opin. Hematol..

[20] Offermanns S. (2006). Activation of platelet function through G protein-coupled receptors. Circ. Res..

[21] Nonne C., Lenain N., Hechler B., Mangin P., Cazenave J., Gachet C., Lanza F. (2005). Importance of platelet phospholipase Cgamma2 signaling in arterial thrombosis as a function of lesion severity. Arterioscler. Thromb. Vasc. Biol..

[22] Bill C.A., Vines C.M. (2020). Phospholipase C. Advances in Experimental Medicine and Biology.

[23] Clemetson K. (2012). Platelets and primary haemostasis. Thromb. Res..

[24] Tomaiuolo M., Brass L.F., Stalker T.J. (2017). Regulation of platelet activation and coagulation and its role in vascular injury and arterial thrombosis. Interv. Cardiol. Clin..

[25] Scridon A. (2022). Platelets and Their Role in Hemostasis and Thrombosis—From Physiology to Pathophysiology and Therapeutic Implications. Int. J. Mol. Sci..

[26] Ma Y.Q., Qin J., Plow E.F. (2007). Platelet integrin αIIbβ3: Activation mechanisms. J. Thromb. Haemost..

[27] Coxon C.H., Geer M.J., Senis Y.A. (2017). ITIM receptors: More than just inhibitors of platelet activation. Blood.

[28] Mazharian A., Senis Y.A. (2025). Defining and Harnessing the Megakaryocyte/Platelet Checkpoint. Mol. Cell. Biol..

[29] Makhoul S., Trabold K., Gambaryan S., Tenzer S., Pillitteri D., Walter U., Jurk K., Makhoul S., Trabold K., Gambaryan S. (2019). cAMP- and cGMP-elevating agents inhibit GPIbα-mediated aggregation but not GPIbα-stimulated Syk activation in human platelets. Cell Commun. Signal..

[30] Murata T., Ushikubi F., Matsuoka T., Hirata M., Yamasaki A., Sugimoto Y., Ichikawa A., Aze Y., Tanaka T., Yoshida N. (1997). Altered pain perception and inflammatory response in mice lacking prostacyclin receptor. Nature.

[31] Ledent C., Vaugeois J.-M., NSchiffmann S., Pedrazzini T., Yacoubi M.E., Van der haeghen J.-J., Costentin J., Heath J.K., Vassart G., Parmentier M. (1997). Aggressiveness, hypoalgesia and high blood pressure in mice lacking the adenosine A2a receptor. Nature.

[32] Yang D., Chen H., Koupenova M., Carroll S.H., Eliades A., Freedman J., Toselli P., Ravid K. (2010). A new role for the A2b adenosine receptor in regulating platelet function. J. Thromb. Haemost..

[33] Hiratsuka T., Sano T., Kato H., Komatsu N., Imajo M., Kamioka Y., Sumiyama K., Banno F., Miyata T., Matsuda M. (2017). Live imaging of extracellular signal-regulated kinase and protein kinase A activities during thrombus formation in mice expressing biosensors based on Förster resonance energy transfer. J. Thromb. Haemost..

[34] Wen L., Feil S., Wolters M., Thunemann M., Regler F., Schmidt K., Friebe A., Olbrich M., Langer H., Gawaz M. (2018). A shear-dependent NO-cGMP-cGKI cascade in platelets acts as an auto-regulatory brake of thrombosis. Nat. Commun..

[35] Webb B., Cheah L., Khalil J., Hindle M., McKay M., Turner N., Kearney M., Ariens R., Duval C., Naseem K. (2025). The critical role of platelet adenylyl cyclase 6 in hemostasis and thrombosis. J. Thromb. Haemost..

[36] Dunkern T.R., Hatzelmann A. (2005). The effect of Sildenafil on human platelet secretory function is controlled by a complex interplay between phosphodiesterases 2, 3 and 5. Cell. Signal..

[37] Hochuli R., Dicenta V., Laspa Z., Sigle M., Harm T., Castor T., Rohlfing A.-K., Gawaz M.P. (2025). Effect of phosphodiesterase inhibitors on platelet function. Biochem. Biophys. Rep..

[38] Hunter R.W., Mackintosh C., Hers I. (2009). Protein kinase C-mediated phosphorylation and activation of PDE3A regulate cAMP levels in human platelets. J. Biol. Chem..

[39] Raslan Z., Naseem K.M. (2014). The control of blood platelets by cAMP signalling. Biochem. Soc. Trans..

[40] Gold M.G. (2019). Swimming regulations for protein kinase A catalytic subunit. Biochem. Soc. Trans..

[41] Aye T.T., Scholten A., Taouatas N., Varro A., Veen T.A.B.V., Vos M.A., Heck A.J.R. (2010). Proteome-wide protein concentrations in the human heart. Mol. Biosyst..

[42] Walker-Gray R., Stengel F., Gold M.G., Walker-Gray R., Stengel F., Gold M.G. (2017). Mechanisms for restraining cAMP-dependent protein kinase revealed by subunit quantitation and cross-linking approaches. Proc. Natl. Acad. Sci. USA.

[43] Huang J., Swieringa F., Solari F.A., Provenzale I., Grassi L., Simone I.D., Baaten C.C.F.M.J., Cavill R., Sickmann A., Frontini M. (2021). Assessment of a complete and classified platelet proteome from genome-wide transcripts of human platelets and megakaryocytes covering platelet functions. Sci. Rep..

[44] Gambaryan S., Kobsar A., Rukoyatkina N., Herterich S., Geiger J., Smolenski A., Lohmann S.M., Walter U. (2010). Thrombin and Collagen Induce a Feedback Inhibitory Signaling Pathway in Platelets Involving Dissociation of the Catalytic Subunit of Protein Kinase A from an NFκB-IκB Complex. J. Biol. Chem..

[45] Bock A., Annibale P., Konrad C., Hannawacker A., Anton S.E., Maiellaro I., Zabel U., Sivaramakrishnan S., Falcke M., Lohse M.J. (2020). Optical Mapping of cAMP Signaling at the Nanometer Scale. Cell.

[46] Pool E., Glebov-McCloud A., Lee L., Hardy J., Pane V., Herberg F., Taylor S., Mehta S., Strack S., Zhang Z. (2025). Aberrant phase separation of two PKA RIβ neurological disorder mutants leads to mechanistically distinct signaling deficits. Cell Rep..

[47] Zhang J.Z., Lu T.-W., Stolerman L.M., Tenner B., Yang J.R., Zhang J.-F., Falcke M., Rangamani P., Taylor S.S., Mehta S. (2020). Phase Separation of a PKA Regulatory Subunit Controls cAMP Compartmentation and Oncogenic Signaling. Cell.

[48] Isensee J., Kaufholz M., Knape M.J., Hasenauer J., Hammerich H., Gonczarowska-Jorge H., Zahedi R.P., Schwede F., Herberg F.W., Hucho T. (2018). PKA-RII subunit phosphorylation precedes activation by cAMP and regulates activity termination. J. Cell Biol..

[49] Gegenbauer K., Nagy Z., Smolenski A. (2013). Cyclic Nucleotide Dependent Dephosphorylation of Regulator of G-Protein Signaling 18 in Human Platelets. PLoS ONE.

[50] O’Donoghue L., Smolenski A. (2024). Roles of G proteins and their GTPase-activating proteins in platelets. Biosci. Rep..

[51] Nagy Z., Smolenski A. (2018). Cyclic nucleotide-dependent inhibitory signaling interweaves with activating pathways to determine platelet responses. Res. Pract. Thromb. Haemost..

[52] Lee D.-H., Kim H.-H., Cho H.-J., Bae J.-S., Yu Y.-B., Park H.-J. (2014). Antiplatelet Effects of Caffeic Acid Due to Ca^2+^ MobilizationInhibition Via cAMP-Dependent Inositol-1, 4, 5-Trisphosphate Receptor Phosphorylation. J. Atheroscler. Thromb..

[53] Butt E., Gambaryan S., Göttfert N., Galler A., Marcus K., Meyer H.E. (2003). Actin Binding of Human LIM and SH3 Protein Is Regulated by cGMP- and cAMP-dependent Protein Kinase Phosphorylation on Serine 146. J. Biol. Chem..

[54] Comer S., Nagy Z., Bolado A., von Kriegsheim A., Gambaryan S., Walter U., Pagel O., Zahedi R., Jurk K., Smolensk I.A. (2020). The RhoA regulators Myo9b and GEF-H1 are targets of cyclic nucleotide-dependent kinases in platelets. J. Thromb. Haemost..

[55] Nagy Z., Wynne K., Kriegsheim A.V., Gambaryan S., Smolenski A. (2015). Cyclic Nucleotide-dependent Protein Kinases Target ARHGAP17 and ARHGEF6 Complexes in Platelets. J. Biol. Chem..

[56] Aburima A., Walladbegi K., Wake J.D., Naseem K.M. (2017). cGMP signaling inhibits platelet shape change through regulation of the RhoA-Rho Kinase-MLC phosphatase signaling pathway. J. Thromb. Haemost..

[57] Murányi A., MacDonald J.A., Deng J.T., Wilson D.P., Haystead T.A.J., Walsh M.P., Erdodi F., Kiss E., Wu Y., Hartshorne D.J. (2002). Phosphorylation of the myosin phosphatase target subunit by integrin-linked kinase. Biochem. J..

[58] Kumm E.J., Pagel O., Gambaryan S., Walter U., Zahedi R.P., Smolenski A., Jurk K. (2020). The Cell Cycle Checkpoint System MAST(L)-ENSA/ARPP19-PP2A is Targeted by cAMP/PKA and cGMP/PKG in Anucleate Human Platelets. Cells.

[59] Beck F., Geiger J., Gambaryan S., Veit J., Vaudel M., Nollau P., Kohlbacher O., Martens L., Walter U., Sickmann A. (2014). Time-resolved characterization of cAMP/PKA-dependent signaling reveals that platelet inhibition is a concerted process involving multiple signaling pathways. Blood.

[60] Pidoux G., Taskén K. (2010). Specificity and spatial dynamics of protein kinase A signaling organized by A-kinase-anchoring proteins. J. Mol. Endocrinol..

[61] Bucko P.J., Scott J.D. (2021). Drugs That Regulate Local Cell Signaling: AKAP Targeting as a Therapeutic Option. Annu. Rev. Pharmacol. Toxicol..

[62] Ercu M., Klussmann E. (2018). Roles of A-Kinase Anchoring Proteins and Phosphodiesterases in the Cardiovascular System. J. Cardiovasc. Dev. Dis..

[63] Kinderman F.S., Kim C., Daake S.V., Ma Y., Pham B.Q., Spraggon G., Xuong N.-H., Jennings P.A., Taylor S.S. (2006). A dynamic mechanism for AKAP binding to RII isoforms of cAMP-dependent protein kinase. Mol. Cell.

[64] Gold M.G., Lygren B., Dokurno P., Hoshi N., McConnachie G., Taskén K., Carlson C.R., Scott J.D., Barford D. (2006). Molecular Basis of AKAP Specificity for PKA Regulatory Subunits. Mol. Cell.

[65] Burgers P.P., van der Heyden M.A.G., Kok B., Heck A.J.R., Scholten A. (2015). A Systematic Evaluation of Protein Kinase A–A-Kinase Anchoring Protein Interaction Motifs. Biochemistry.

[66] Falcone J.I., Cleveland K.H., Kang M., Odle B.J., Forbush K.A., Scott J.D. (2025). The evolution of AKAPs and emergence of PKA isotype selective anchoring determinants. J. Biol. Chem..

[67] Dema A., Perets E., Schulz M.S., Deák V.A., Klussmann E. (2015). Pharmacological targeting of AKAP-directed compartmentalized cAMP signalling. Cell. Signal..

[68] Subramanian H., Nikolaev V.O., Subramanian H., Nikolaev V.O. (2023). A-Kinase Anchoring Proteins in Cardiac Myocytes and Their Roles in Regulating Calcium Cycling. Cells.

[69] Gold M.G., Gonen T., Scott J.D. (2013). Local cAMP signaling in disease at a glance. J. Cell Sci..

[70] Margarucci L., Roest M., Preisinger C., Bleijerveld O.B., Holten T.C.V., Heck A.J.R., Scholten A. (2011). Collagen stimulation of platelets induces a rapid spatial response of cAMP and cGMP signaling scaffolds. Mol. Biosyst..

[71] Petrich B., Marchese P., Ruggeri Z., Spiess S., Weichert P., Ye F., Tiedt R., Skoda R., Monkley S., Critchley D. (2007). Talin is required for integrin-mediated platelet function in hemostasis and thrombosis. J. Exp. Med..

[72] Venkatesh B., Golla K., Hong F., Haage A., Kim H., Tanentzapf G. (2025). Talin autoinhibition is required for normal hemostasis. Platelets.

[73] Lagarrigue F., Paul D.S., Gingras A.R., Valadez A.J., Sun H., Lin J., Cuevas M.N., Ablack J.N., Lopez-Ramirez M.A., Bergmeier W. (2020). Talin-1 is the principal platelet Rap1 effector of integrin activation. Blood.

[74] Nieswandt B., Moser M., Pleines I., Varga-Szabo D., Monkley S., Critchley D., Fässler R. (2007). Loss of talin1 in platelets abrogates integrin activation, platelet aggregation, and thrombus formation in vitro and in vivo. J. Exp. Med..

[75] Raslan Z., Magwenzi S., Aburima A., Taskén K., Naseem K. (2015). Targeting of type I protein kinase A to lipid rafts is required for platelet inhibition by the 3′,5′-cyclic adenosine monophosphate-signaling pathway. J. Thromb. Haemost..

[76] Burgers P.P., Ma Y., Margarucci L., Mackey M., van der Heyden M.A.G., Ellisman M., Scholten A., Taylor S.S., Heck A.J.R. (2012). A Small Novel A-Kinase Anchoring Protein (AKAP) That Localizes Specifically Protein Kinase A-Regulatory Subunit I (PKA-RI) to the Plasma Membrane. J. Biol. Chem..

[77] Burgers P.P., Bruystens J., Burnley R.J., Nikolaev V.O., Keshwani M., Wu J., Janssen B.J.C., Taylor S.S., Heck A.J.R., Scholten A. (2016). Structure of smAKAP and its regulation by PKA-mediated phosphorylation. FEBS J..

[78] Schoenwaelder S.M., Ono A., Sturgeon S., Chan S.M., Mangin P., Maxwell M.J., Turnbull S., Mulchandani M., Anderson K., Kauffenstein G. (2007). Identification of a unique co-operative phosphoinositide 3-kinase signaling mechanism regulating integrin alpha IIb beta 3 adhesive function in platelets. J. Biol. Chem..

[79] Guidetti G.F., Canobbio I., Torti M. (2015). PI3K/Akt in platelet integrin signaling and implications in thrombosis. Adv. Biol. Regul..

[80] Nuytens K., Tuand K., Michele M.D., Boonen K., Waelkens E., Freson K., Creemers J. (2013). Platelets of mice heterozygous for neurobeachin, a candidate gene for autism spectrum disorder, display protein changes related to aberrant protein kinase A activity. Mol. Autism.

[81] Oda A., Miki H., Wada I., Yamaguchi H., Yamazaki D., Suetsugu S., Nakajima M., Nakayama A., Okawa K., Miyazaki H. (2005). WAVE/Scars in platelets. Blood.

[82] Calaminus S.D.J., McCarty O.J.T., Auger J.M., Pearce A.C., Insall R.H., Watson S.P., Machesky L.M. (2007). A major role for Scar/WAVE-1 downstream of GPVI in platelets. J. Thromb. Haemost..

[83] Khalil J.S., Law R., Raslan Z., Cheah L.T., Hindle M.S., Aburima A.A., Kearney M.T., Naseem K.M. (2024). Protein Kinase A Regulates Platelet Phosphodiesterase 3A through an A-Kinase Anchoring Protein Dependent Manner. Cells.

[84] Moser M., Legate K.R., Zent R., Fässler R. (2009). The tail of integrins, talin, and kindlins. Science.

[85] Dedden D., Schumacher S., Kelley C.F., Zacharias M., Biertümpfel C., Fässler R., Mizuno N. (2019). The Architecture of Talin1 Reveals an Autoinhibition Mechanism. Cell.

[86] Zhu L., Yang J., Bromberger T., Holly A., Lu F., Liu H., Sun K., Klapproth S., Hirbawi J., Byzova T.V. (2017). Structure of Rap1b bound to talin reveals a pathway for triggering integrin activation. Nat. Commun..

[87] Kang M., Otani Y., Guo Y., Yan J., Goult B.T., Howe A.K., Kang M., Otani Y., Guo Y., Yan J. (2024). The focal adhesion protein talin is a mechanically gated A-kinase anchoring protein. Proc. Natl. Acad. Sci. USA.

[88] Guidetti G.F., Manganaro D., Consonni A., Canobbio I., Balduini C., Torti M. (2013). Phosphorylation of the guanine-nucleotide-exchange factor CalDAG-GEFI by protein kinase A regulates Ca2+-dependent activation of platelet Rap1b GTPase. Biochem. J..

[89] Schultess J., Danielewski O., Smolenski A.P. (2005). Rap1GAP2 is a new GTPase-activating protein of Rap1 expressed in human platelets. Blood.

[90] McClatchey A.I. (2014). ERM proteins at a glance. J. Cell Sci..

[91] Gamulescu M., Seifert K., Tingart M., Falet H., Hoffmeister K. (2003). Platelet moesin interacts with PECAM-1 (CD31). Platelets.

[92] Nakamura F., Huang L., Pestonjamasp K., Luna E.J., Furthmayr H. (1999). Regulation of F-actin binding to platelet moesin in vitro by both phosphorylation of threonine 558 and polyphosphatidylinositides. Mol. Biol. Cell.

[93] Dransfield D.T., Bradford A.J., Smith J., Martin M., Roy C., Mangeat P.H., Goldenring J.R. (1997). Ezrin is a cyclic AMP-dependent protein kinase anchoring protein. EMBO J..

[94] Ruppelt A., Mosenden R., Grönholm M., Aandahl E.M., Tobin D.T., Carlson C.R., Abrahamsen H., Herberg F.W., Carpén O., Taskén K. (2007). Inhibition of T cell activation by cyclic adenosine 5′-monophosphate requires lipid raft targeting of protein kinase A type I by the A-kinase anchoring protein ezrin. J. Immunol..

[95] Deming P.B., Campbell S.L., Stone J.B., Rivard R.L., Mercier A.L., Howe A.K. (2015). Anchoring of Protein Kinase A by ERM (Ezrin-Radixin-Moesin) Proteins Is Required for Proper Netrin Signaling through DCC (Deleted in Colorectal Cancer). J. Biol. Chem..

[96] Dukic A.R., Haugen L.H., Pidoux G., Leithe E., Bakke O., Taskén K. (2017). A protein kinase A-ezrin complex regulates connexin 43 gap junction communication in liver epithelial cells. Cell. Signal..

[97] Javed E., Nayak A., Jannu A., Cohen A., Dewes I., Wang R., Tang D., Deshpande D., Penn R. (2025). A-Kinase-Anchoring Protein Subtypes Differentially Regulate GPCR Signaling and Function in Human Airway Smooth Muscle. Am. J. Respir. Cell Mol. Biol..

[98] Leiphrakpam P.D., Brattain M.G., Black J.D., Wang J. (2018). TGFβ and IGF1R signaling activates protein kinase A through differential regulation of ezrin phosphorylation in colon cancer cells. J. Biol. Chem..

[99] Perino A., Ghigo A., Ferrero E., Morello F., Santulli G., Baillie G., Damilano F., Dunlop A.J., Pawson C., Walser R. (2011). Integrating cardiac PIP3 and cAMP signaling through a PKA anchoring function of p110γ. Mol. Cell.

[100] Ghigo A., Perino A., Mehel H., Zahradníková A., Morello F., Leroy J., Nikolaev V., Damilano F., Cimino J., Luca E.D. (2012). Phosphoinositide 3-kinase γ protects against catecholamine-induced ventricular arrhythmia through protein kinase A-mediated regulation of distinct phosphodiesterases. Circulation.

[101] Ghigo A., Murabito A., Sala V., Pisano A.R., Bertolini S., Gianotti A., Caci E., Montresor A., Premchandar A., Pirozzi F. (2022). A PI3Kγ mimetic peptide triggers CFTR gating, bronchodilation, and reduced inflammation in obstructive airway diseases. Sci. Transl. Med..

[102] Ribes A., Oprescu A., Viaud J., Hnia K., Chicanne G., Xuereb J.-M., Severin S., Gratacap M.-P., Payrastre B. (2020). Phosphoinositide 3-kinases in platelets, thrombosis and therapeutics. Biochem. J..

[103] Durrant T.N., Hers I. (2020). PI3K inhibitors in thrombosis and cardiovascular disease. Clin. Transl. Med..

[104] Curto M., McClatchey A.I. (2008). Nf2/Merlin: A coordinator of receptor signalling and intercellular contact. Br. J. Cancer.

[105] Sekido Y., Sato T. (2023). NF2 alteration in mesothelioma. Front. Toxicol..

[106] Grönholm M., Vossebein L., Carlson C.R., Kuja-Panula J., Teesalu T., Alfthan K., Vaheri A., Rauvala H., Herberg F.W., Taskén K. (2003). Merlin links to the cAMP neuronal signaling pathway by anchoring the RIbeta subunit of protein kinase A. J. Biol. Chem..

[107] Penny C.J., Gold M.G. (2018). Mechanisms for localising calcineurin and CaMKII in dendritic spines. Cell. Signal..

[108] Murphy J.G., Sanderson J.L., Gorski J.A., Scott J.D., Catterall W.A., Sather W.A., Dell’Acqua M.L. (2014). AKAP-anchored PKA maintains neuronal L-type calcium channel activity and NFAT transcriptional signaling. Cell Rep..

[109] Simmons S.C., Flerlage W.J., Langlois L.D., Shepard R.D., Bouslog C., Thomas E.H., Gouty K.M., Sanderson J.L., Gouty S., Cox B.M. (2024). AKAP150-anchored PKA regulates synaptic transmission and plasticity, neuronal excitability and CRF neuromodulation in the mouse lateral habenula. Commun. Biol..

[110] Church T.W., Tewatia P., Hannan S., Antunes J., Eriksson O., Smart T.G., Kotaleski J.H., Gold M.G. (2021). AKAP79 enables calcineurin to directly suppress protein kinase A activity. eLife.

[111] Nichols C.B., Rossow C.F., Navedo M.F., Westenbroek R.E., Catterall W.A., Santana L.F., McKnight G.S. (2010). Sympathetic Stimulation of Adult Cardiomyocytes Requires Association of AKAP5 with a Subpopulation of L-Type Calcium Channels. Circ. Res..

[112] Qasim H., McConnell B.K. (2020). AKAP12 Signaling Complex: Impacts of Compartmentalizing cAMP-Dependent Signaling Pathways in the Heart and Various Signaling Systems. J. Am. Heart Assoc..

[113] Smith M.R., Costa G. (2024). Insights into the regulation of mRNA translation by scaffolding proteins. Biochem. Soc. Trans..

[114] Repetto D., Brockhaus J., Rhee H.J., Lee C., Kilimann M.W., Rhee J., Northoff L.M., Guo W., Reissner C., Missler M. (2018). Molecular Dissection of Neurobeachin Function at Excitatory Synapses. Front. Synaptic Neurosci..

[115] Lützenkirchen F.P., Zhu Y., Maric H.M., Boeck D.S., Gromova K.V., Kneussel M., Lützenkirchen F.P., Zhu Y., Maric H.M., Boeck D.S. (2024). Neurobeachin regulates receptor downscaling at GABAergic inhibitory synapses in a protein kinase A-dependent manner. Commun. Biol..

[116] Alto N.M., Soderling J., Scott J.D. (2002). Rab32 is an A-kinase anchoring protein and participates in mitochondrial dynamics. J. Cell Biol..

[117] Bui M., Gilady S.Y., Fitzsimmons R.E.B., Benson M.D., Lynes E.M., Gesson K., Alto N.M., Strack S., Scott J.D., Simmen T. (2010). Rab32 Modulates Apoptosis Onset and Mitochondria-associated Membrane (MAM) Properties. J. Biol. Chem..

[118] Johnson K.M., Marley M.G., Drizyte-Miller K., Chen J., Cao H., Mostafa N., Schott M.B., McNiven M.A., Razidlo G.L. (2025). Rab32 regulates Golgi structure and cell migration through Protein Kinase A-mediated phosphorylation of Optineurin. Proc. Natl. Acad. Sci. USA.

[119] Drizyte-Miller K., Chen J., Cao H., Schott M.B., McNiven M.A. (2020). The small GTPase Rab32 resides on lysosomes to regulate mTORC1 signaling. J. Cell Sci..

[120] Ambrosio A.L., Boyle J.A., Pietro S.M.D. (2012). Mechanism of platelet dense granule biogenesis: Study of cargo transport and function of Rab32 and Rab38 in a model system. Blood.

[121] Huang L.J.-s., Durick K., Weiner J.A., Chun J., Taylor S.S. (1997). D-AKAP2, a novel protein kinase A anchoring protein with a putative RGS domain. Proc. Natl. Acad. Sci. USA.

[122] Sarma G., Moody I., Ilouz R., Phan R., Sankaran B., Hall R., Taylor S. (2015). D-AKAP2:PKA RII:PDZK1 ternary complex structure: Insights from the nucleation of a polyvalent scaffold. Protein Sci..

[123] Eggers C.T., Schafer J.C., Goldenring J.R., Taylor S.S. (2009). D-AKAP2 interacts with Rab4 and Rab11 through its RGS domains and regulates transferrin receptor recycling. J. Biol. Chem..

[124] Sorvina A., Shandala T., Brooks D.A. (2016). Drosophila Pkaap regulates Rab4/Rab11-dependent traffic and Rab11 exocytosis of innate immune cargo. Biol. Open.

[125] Sherpa R.T., Moshal K.S., Agarwal S.R., Ostrom R.S., Harvey R.D. (2024). Role of protein kinase A and A kinase anchoring proteins in buffering and compartmentation of cAMP signalling in human airway smooth muscle cells. Br. J. Pharmacol..

[126] Shirakawa R., Yoshioka A., Horiuchi H., Nishioka H., Tabuchi A., Kita T. (2000). Small GTPase Rab4 regulates Ca^2+^-induced alpha-granule secretion in platelets. J. Biol. Chem..

[127] Segura-Roman A., Citron Y., Shin M., Sindoni N., Maya-Romero A., Rapp S., Goul C., Mancias J., Zoncu R. (2025). Autophagosomes anchor an AKAP11-dependent regulatory checkpoint that shapes neuronal PKA signaling. EMBO J..

[128] Logue J.S., Whiting J.L., Tunquist B., Sacks D.B., Langeberg L.K., Wordeman L., Scott J.D. (2011). AKAP220 protein organizes signaling elements that impact cell migration. J. Biol. Chem..

[129] Stalder D., Yakunin I., Pereira C., Eden J., Gershlick D.C. (2024). Recruitment of PI4KIIIβ to the Golgi by ACBD3 is dependent on an upstream pathway of a SNARE complex and golgins. Mol. Biol. Cell.

[130] Yue X., Qian Y., Gim B., Lee I., Yue X., Qian Y., Gim B., Lee I. (2019). Acyl-CoA-Binding Domain-Containing 3 (ACBD3; PAP7; GCP60): A Multi-Functional Membrane Domain Organizer. Int. J. Mol. Sci..

[131] Liu J., Li H., Papadopoulos V. (2003). PAP7, a PBR/PKA-RIalpha-associated protein: A new element in the relay of the hormonal induction of steroidogenesis. J. Steroid Biochem. Mol. Biol..

[132] Sherpa R., Fiore C., Moshal K., Wadsworth A., Rudokas M., Agarwal S., Harvey R. (2021). Mitochondrial A-kinase anchoring proteins in cardiac ventricular myocytes. Physiol. Rep..

[133] Walton K., Leier A., Sztul E. (2020). Regulating the regulators: Role of phosphorylation in modulating the function of the GBF1/BIG family of Sec7 ARF-GEFs. FEBS Lett..

[134] Li H., Adamik R., Pacheco-Rodriguez G., Moss J., Vaughan M. (2003). Protein kinase A-anchoring (AKAP) domains in brefeldin A-inhibited guanine nucleotide-exchange protein 2 (BIG2). Proc. Natl. Acad. Sci. USA.

[135] Islam A., Jones H., Hiroi T., Lam J., Zhang J., Moss J., Vaughan M., Levine S.J. (2008). cAMP-dependent protein kinase A (PKA) signaling induces TNFR1 exosome-like vesicle release via anchoring of PKA regulatory subunit RIIbeta to BIG2. J. Biol. Chem..

[136] Puxeddu E., Uhart M., Li C.-C., Ahmad F., Pacheco-Rodriguez G., Manganiello V.C., Moss J., Vaughan M., Puxeddu E., Uhart M. (2009). Interaction of phosphodiesterase 3A with brefeldin A-inhibited guanine nucleotide-exchange proteins BIG1 and BIG2 and effect on ARF1 activity. Proc. Natl. Acad. Sci. USA.

[137] Kuroda F., Moss J., Vaughan M. (2007). Regulation of brefeldin A-inhibited guanine nucleotide-exchange protein 1 (BIG1) and BIG2 activity via PKA and protein phosphatase 1gamma. Proc. Natl. Acad. Sci. USA.

[138] Hong E.-H., Kim J.-Y., Kim J.-H., Lim D.-S., Kim M., Kim J.-Y., Hong E.-H., Kim J.-Y., Kim J.-H., Lim D.-S. (2018). BIG2-ARF1-RhoA-mDia1 Signaling Regulates Dendritic Golgi Polarization in Hippocampal Neurons. Mol. Neurobiol..

[139] Johnson K.R., Nicodemus-Johnson J., Carnegie G.K., Danziger R.S. (2012). Molecular evolution of a-kinase anchoring protein (AKAP)-7: Implications in comparative PKA compartmentalization. BMC Evol. Biol..

[140] Jones B.W., Brunet S., Gilbert M.L., Nichols C.B., Su T., Westenbroek R.E., Scott J.D., Catterall W.A., McKnight G.S., Jones B.W. (2012). Cardiomyocytes from AKAP7 knockout mice respond normally to adrenergic stimulation. Proc. Natl. Acad. Sci. USA.

[141] Park T., Forbush K., Li Y., Vivas O., Rosenthal K.J., Falcone J., Wong C.J., Bruce J.E., Moreno C., Dessauer C.W. (2025). Long AKAP18 isoforms anchor ubiquitin specific proteinases and coordinate calcium reuptake at the sarcoplasmic reticulum. J. Biol. Chem..

[142] Singh A., Rigatti M., Le A.V., Carlson C.R., Moraru I.I., Dodge-Kafka K.L. (2015). Analysis of AKAP7γ Dimerization. J. Signal Transduct..

[143] Lin R.-Y., Moss S.B., Rubin C.S. (1995). Characterization of S-AKAP84, a Novel Developmentally Regulated A Kinase Anchor Protein of Male Germ Cells. J. Biol. Chem..

[144] Huang L.J.-S., Durick K., Weiner J.A., Chun J., Taylor S.S. (1997). Identification of a novel protein kinase A anchoring protein that binds both type I and type II regulatory subunits. J. Biol. Chem..

[145] Ma Y., Taylor S. (2002). A 15-Residue Bifunctional Element in d-AKAP1 Is Required for Both Endoplasmic Reticulum and Mitochondrial Targeting. J. Biol. Chem..

[146] Huang L.J.-S., Wang L., Ma Y., Durick K., Perkins G., Deerinck T.J., Ellisman M.H., Taylor S.S. (1999). NH2-Terminal Targeting Motifs Direct Dual Specificity A-Kinase–anchoring Protein 1 (D-AKAP1) to Either Mitochondria or Endoplasmic Reticulum. J. Cell Biol..

[147] Chaudhry A., Zhang C., Granneman J.G. (2002). Characterization of RII(beta) and D-AKAP1 in differentiated adipocytes. Am. J. Physiol. Cell Physiol..

[148] Meiri D., Greeve M., Brunet A., Finan D., Wells C., LaRose J., Rottapel R. (2009). Modulation of Rho guanine exchange factor Lfc activity by protein kinase A-mediated phosphorylation. Mol. Cell. Biol..

[149] Wu S., Li L., Wu X., Wong C.K., Sun F., Cheng C.Y. (2021). AKAP9 supports spermatogenesis through its effects on microtubule and actin cytoskeletons in the rat testis. FASEB J..

[150] Sehrawat S., Ernandez T., Cullere X., Takahashi M., Ono Y., Komarova Y., Mayadas T.N. (2010). AKAP9 regulation of microtubule dynamics promotes Epac1-induced endothelial barrier properties. Blood.

[151] Fu Y., Huang S., Pan R., Chen X., Liu T., Zhang R., Zhu F., Fang Q., Wu L., Dai J. (2025). The PDE4DIP-AKAP9 axis promotes lung cancer growth through modulation of PKA signalling. Commun. Biol..

[152] Burianek L.E., Soderling S.H. (2013). Under lock and key: Spatiotemporal regulation of WASP family proteins coordinates separate dynamic cellular processes. Semin. Cell Dev. Biol..

[153] Alekhina O., Burstein E., Billadeau D.D. (2017). Cellular functions of WASP family proteins at a glance. J. Cell Sci..

[154] Westphal R.S., Soderling S.H., Alto N.M., Langeberg L.K., Scott J.D. (2000). Scar/WAVE-1, a Wiskott–Aldrich syndrome protein, assembles an actin-associated multi-kinase scaffold. EMBO J..

[155] Matt U., Sharif O., Martins R., Furtner T., Langeberg L., Gawish R., Elbau I., Zivkovic A., Lakovits K., Oskolkova O. (2013). WAVE1 mediates suppression of phagocytosis by phospholipid-derived DAMPs. J. Clin. Investig..

[156] Diviani D., Soderling J., Scott J.D. (2001). AKAP-Lbc anchors protein kinase A and nucleates Galpha 12-selective Rho-mediated stress fiber formation. J. Biol. Chem..

[157] Diviani D., Baisamy L., Appert-Collin A. (2006). AKAP-Lbc: A molecular scaffold for the integration of cyclic AMP and Rho transduction pathways. Eur. J. Cell Biol..

[158] Martin J.W., Cavagnini K.S., Brawley D.N., Berkley C.Y., Smolski W.C., Garcia R.D., Towne A.L., Sims J.R., Meigs T.E., Martin J.W. (2016). A Gα12-specific Binding Domain in AKAP-Lbc and p114RhoGEF. J. Mol. Signal..

[159] Delaunay M., Paterek A., Gautschi I., Scherler G., Diviani D. (2024). AKAP2-anchored extracellular signal-regulated kinase 1 (ERK1) regulates cardiac myofibroblast migration. Biochim. Biophys. Acta (BBA)-Mol. Cell Res..

[160] Gold M., Reichow S., O’Neill S., Weisbrod C., Langeberg L., Bruce J., Gonen T., Scott J. (2012). AKAP2 anchors PKA with aquaporin-0 to support ocular lens transparency. EMBO Mol. Med..

[161] Aye T., Mohammed S., Toorn H.V.D., Veen T.V., Heyden M.D., Scholten A., Heck A. (2009). Selectivity in enrichment of cAMP-dependent protein kinase regulatory subunits type I and type II and their interactors using modified cAMP affinity resins. Mol. Cell. Proteom..

[162] Kurosu T., Hernández A.I., Wolk J., Liu J., Schwartz J.H. (2009). α/β-tubulin are A kinase anchor proteins for type I PKA in neurons. Brain Res..

[163] Lyu J., DeMarco A.G., Sweet R.A., Grubisha M.J. (2025). MAP2 phosphorylation: Mechanisms, functional consequences, and emerging insights. Front. Cell. Neurosci..

[164] Theurkauf W.E., Vallee R.B. (1982). molecular characterization of the cAMP-dependent protein kinase bound to microtubule-associated protein 2. J. Biol. Chem..

[165] Lohmann S.M., DeCamilli P., Einig I., Walter U. (1984). High-affinity binding of the regulatory subunit (RII) of cAMP-dependent protein kinase to microtubule-associated and other cellular proteins. Proc. Natl. Acad. Sci. USA.

[166] Zhong H., Sia G.-M., Sato T.R., Gray N.W., Mao T., Khuchua Z., Huganir R.L., Svoboda K. (2009). Subcellular Dynamics of Type II PKA in Neurons. Neuron.

[167] Ashburner M., Ball C., Blake J., Botstein J., Butler H., Cherry J., Davis A., Dolinski K., Dwight S., Eppig J. (2000). Gene ontology: Tool for the unification of biology. The Gene Ontology Consortium. Nat. Genet..

[168] Delaval B., Doxsey S.J. (2010). Pericentrin in cellular function and disease. J. Cell Biol..

[169] Diviani D., Langeberg L.K., Doxsey S.J., Scott J.D. (2000). Pericentrin anchors protein kinase A at the centrosome through a newly identified RII-binding domain. Curr. Biol..

[170] Kolobova E., Roland J.T., Lapierre L.A., Williams J.A., Mason T.A., Goldenring J.R. (2017). The C-terminal region of A-kinase anchor protein 350 (AKAP350A) enables formation of microtubule-nucleation centers and interacts with pericentriolar proteins. J. Biol. Chem..

[171] Russell M.A. (2020). Synemin Redefined: Multiple Binding Partners Results in Multifunctionality. Front. Cell Dev. Biol..

[172] Russell M.A., Lund L.M., Haber R., McKeegan K., Cianciola N., Bond M. (2006). The intermediate filament protein, synemin, is an AKAP in the heart. Arch. Biochem. Biophys..

[173] Kimmerlin Q., Strassel C., Eckly A., Lanza F. (2023). The tubulin code in platelet biogenesis. Semin. Cell Dev. Biol..

[174] Begonja A.J., Gambaryan S., Schulze H., Patel-Hett S., Italiano J.E., Hartwig J.H., Walter U. (2013). Differential roles of cAMP and cGMP in megakaryocyte maturation and platelet biogenesis. Exp. Hematol..

[175] Choi S.R., Blum T.B., Giono M., Roy B., Vakonakis I., Schmid D., Oelgarth N., Ranganathan A., Gossert A.D., Shivashankar G.V. (2026). Structural basis of microtubule-mediated signal transduction. Cell.

[176] Chaturvedi D., Poppleton H., Stringfield T., Barbier A., Patel T. (2006). Subcellular Localization and Biological Actions of Activated RSK1 Are Determined by Its Interactions with Subunits of Cyclic AMP-Dependent Protein Kinase. Mol. Cell. Biol..

[177] Houles T., Roux P.P. (2018). Defining the role of the RSK isoforms in cancer. Semin. Cancer Biol..

[178] Manne B., Münzer P., Badolia R., Walker-Allgaier B., Campbell R., Middleton E., Weyrich A., Kunapuli S., Borst O., Rondina M. (2018). PDK1 governs thromboxane generation and thrombosis in platelets by regulating activation of Raf1 in the MAPK pathway. J. Thromb. Haemost..

[179] Schwarz U.R., Kobsar A.L., Koksch M., Walter U., Eigenthaler M. (2000). Inhibition of agonist-induced p42 and p38 mitogen-activated protein kinase phosphorylation and CD40 ligand/P-selectin expression by cyclic nucleotide-regulated pathways in human platelets. Biochem. Pharmacol..

[180] Fatima A., Hoeber J., Schuster J., Koshimizu E., Maya-Gonzalez C., Keren B., Mignot C., Akram T., Ali Z., Miyatake S. (2021). Monoallelic and bi-allelic variants in NCDN cause neurodevelopmental delay, intellectual disability, and epilepsy. Am. J. Hum. Genet..

[181] Hermann J.S., Skroblin P., Bertinetti D., Hanold L.E., von der Heide E.K., Wagener E.-M., Zenn H.-M., Klussmann E., Kennedy E.J., Herberg F.W. (2015). Neurochondrin is an atypical RIIα-specific A-kinase anchoring protein. Biochim. et Biophys. Acta.

[182] Faulkner E.L., Pike J.A., Garlick E., Neely R.K., Styles I.B., Watson S.P., Poulter N.S., Thomas S.G. (2025). Expansion microscopy allows quantitative characterization of structural organization of platelet aggregates. J. Thromb. Haemost..

[183] Yarman Y., Zhao X., Ahn H., Thomson H.A., Sarkar A., Yuan T., Roberts M.E., Wurtzel J., Diamond S.L., Tesmer J.J.G. (2026). Understanding how a highly prevalent GRK5 polymorphism affects platelets and enhances thrombotic risk. Blood.

[184] Baillie G.S., Tejeda G.S., Kelly M.P., Baillie G.S., Tejeda G.S., Kelly M.P. (2019). Therapeutic targeting of 3′,5′-cyclic nucleotide phosphodiesterases: Inhibition and beyond. Nat. Rev. Drug Discov..

[185] S. G. (2022). The Role of NO/sGC/cGMP/PKG Signaling Pathway in Regulation of Platelet Function. Cells.

[186] Slingsby M.H.L., Nyberg M., Egelund J., Mandrup C.M., Frikke-Schmidt R., Kirkby N.S., Hellsten Y. (2017). Aerobic exercise training lowers platelet reactivity and improves platelet sensitivity to prostacyclin in pre- and postmenopausal women. J. Thromb. Haemost..

[187] O’Donoghue L., Crognale D., Delahunt E., Smolenski A., O’Donoghue L., Crognale D., Delahunt E., Smolenski A. (2024). Effects of exercise on cAMP-mediated platelet inhibition in young women: A pilot study. Eur. J. Appl. Physiol..

[188] Wickham K.A., Nørregaard L.B., Slingsby M.H.L., Cheung S.S., Hellsten Y. (2022). High-Intensity Exercise Training Improves Basal Platelet Prostacyclin Sensitivity and Potentiates the Response to Dual Anti-Platelet Therapy in Postmenopausal Women. Biomolecules.

[189] Gautam D., Goggi G., Battinelli E.M., Gautam D., Goggi G., Battinelli E.M. (2026). Platelet Subpopulations in Health and Disease: Heterogeneity, Clinical Associations, and Therapeutic Targeting. Cells.

[190] Ruseska I., Zimmer A. (2020). Internalization mechanisms of cell-penetrating peptides. Beilstein J. Nanotechnol..

[191] Trory J.S., Vautrinot J., May C.J., Hers I. (2025). PROTACs in platelets: Emerging antithrombotic strategies and future perspectives. Curr. Opin. Hematol..

[192] Soini L., Leysen S., Davis J., Ottmann C. (2022). Molecular glues to stabilise protein–protein interactions. Curr. Opin. Chem. Biol..

[193] King E.A., Meyers M., Nomura D.K., King E.A., Meyers M., Nomura D.K. (2025). Induced proximity-based therapeutic modalities. Nat. Rev. Drug Discov..

